# Massive RNA Editing in Ascetosporean Mitochondria

**DOI:** 10.1264/jsme2.ME24070

**Published:** 2025-03-15

**Authors:** Akinori Yabuki, Chihaya Fujii, Euki Yazaki, Akihiro Tame, Keiko Mizuno, Yumiko Obayashi, Yoshitake Takao

**Affiliations:** 1 Japan Agency for Marine-Earth Science and Technology, Yokosuka, Kanagawa 237–0061, Japan; 2 Graduate School of Agricultural Science, Tohoku University, Sendai, Miyagi 980–8572 Japan; 3 Advanced Institute for Marine Ecosystem Change (WPI-AIMEC), Yokohama, Kanagawa 236–0001, Japan; 4 Research Center for Advanced Analysis, National Agriculture and Food Research Organization, Tsukuba, 305–8518 Japan; 5 Marine Works Japan Ltd., Yokosuka, Kanagawa 237–0063, Japan; 6 Center for Marine Environmental Studies, Ehime University, Matsuyama, Ehime 790–8577, Japan; 7 Department of Marine Science and Technology, Faculty of Marine Science and Technology, Fukui Prefecture University, Obama, Fukui 917–0003, Japan

**Keywords:** ADAR, mitochondria, PPR-DYW protein, protists, RNA editing

## Abstract

Ascetosporeans are parasitic protists of invertebrates. A deep sequencing ana­lysis of species within the orders Mikrocytida, Paramyxida, and Haplosporida using metagenomic approaches revealed that their mitochondria were functionally reduced and their organellar genomes were lacking. Ascetosporeans belonging to the order Paradinida have not been sequenced, and the nature of their mitochondria remains unclear. We herein established two cultures of Paradinida and conducted DNA and RNA sequencing ana­lyses. The results obtained indicate that mitochondrial function in paradinids was not reduced and their organellar genomes were retained. In contrast, their mitochondrial genomes were involved in massive A-to-I and C-to-U substitution types of RNA editing. All edits in protein-coding genes were nonsynonymous substitutions, and likely had a restorative function against negative mutations. Furthermore, we detected possible sequences of DYW type of pentatricopeptide repeat (PPR-DYW) protein and a homologue of adenosine deaminase acting on RNA (ADAR-like), which are key enzymes for C-to-U and A-to-I substitutions, respectively. An immunofluorescence ana­lysis showed that ADAR-like of paradinids may specifically localize within mitochondria. These results expand our knowledge of the diversity and complexity of organellar RNA editing phenomena.

Ascetosporea (ascetosporeans) is a class of Endomyxa, Rhizaria, and all species are parasites of invertebrates. Their cell cultures have not been reported, and the complete life cycle of ascetosporeans remains unclear (Bass *et al.*, 2019). Since some ascetosporeans cause serious damage to aquaculture, they are important targets in fishery sciences (Mourton *et al.*, 1992; Hartikainen *et al.*, 2014a, 2014b). Five orders in Ascetosporea have been proposed to date. Several species from three orders, *i.e.*, Mikrocytida, Paramyxida, and Haplosporida, have been sequenced in detail and analyzed through metagenomic approaches using infected host organisms collected from natural habitats (Burki *et al.*, 2013; Onuț-Brännström *et al.*, 2023; Hiltunen Thorén *et al.*, 2024). Previous studies showed that the mitochondria of Mikrocytida were functionally reduced to mitochondrion-related organelles (MROs), and their organellar genomes were not observed (Burki *et al.*, 2013; Onuț-Brännström *et al.*, 2023). Although mitochondria-like structures have been detected in Paramyxida (Perkins, 1976; Villalba *et al.*, 1993), their mitochondrial genomes have not been identified by mole­cular studies (Hiltunen Thorén *et al.*, 2024) and their functional nature remains unclear. In contrast, a possible mitochondrial genome fragment has been reported from *Bonamia ostreae* (Order Haplosporida), and the genes encoding the subunits of ATPase, cytochrome *c* oxidase, and cytochrome *b*, ribosomal RNAs, and transfer RNAs have been identified (Hiltunen Thorén *et al.*, 2024). The two other orders, Paradinida and Claustrosporida, have not yet been sequenced in detail, and the function and evolutionary state of their mitochondria have not been elucidated in detail.

RNA editing is an important cellular process that results in RNA modifications. Several types of RNA editing have been reported and play important roles in changing functional proteins and non-coding RNA (Gott and Emeson, 2000). The adenosine-to-inosine (A-to-I) substitution mediated by adenosine deaminase acting on RNA (ADAR) in the metazoan nucleus and the cytidine-to-uridine (C-to-U) substitution mediated by DYW type of pentatricopeptide repeat (PPR-DYW) protein in plant organelles are the most studied forms of RNA editing (Shikanai, 2015; Eisenberg and Levanon, 2018). PPR-DYW protein has also been detected in protists that similarly possess C-to-U substitutions in their mitochondria, which may have resulted from multiple horizontal transfers from plants (Knoop and Rüdinger, 2010; Schallenberg-Rüdinger *et al.*, 2013). In contrast, it is widely accepted that ADAR is unique to metazoans (Gott and Emeson, 2000), although a homologue of ADAR (ADAR-like) has been reported from the protist *Symbiodinium* spp. (Liew *et al.*, 2017). Since fungi and early-branching opisthokonts lack ADAR in their genomes, ADAR is considered to have evolved from adenosine deaminase acting on tRNA (ADAT), which is conserved in all eukaryotes, in the metazoan ancestor (Gott and Emeson, 2000).

In the present study, we established clonal cultures of Paradinida and sequenced their mitochondrial genomes. RNA-seq analyses showed that the A-to-I and C-to-U substitution types of RNA editing occurred within their mitochondria. Furthermore, PPR-DYW protein and ADAR-like were both detected, and the subcellular localization of ADAR-like in mitochondria was suggested. These results indicate that paradinids utilize both plant-type and metazoan-type RNA editing systems in their mitochondria. This unique editing system demonstrates the diversity and flexibility of cell function.

## Materials and Methods

### Sample acquisition, culturing, and microscopy

Water samples were collected from Tokyo and Suruga Bays (Table S1). A small aliquot of each sample was added to Hemi medium (Tashyreva *et al.*, 2018) supplemented with a 5‍ ‍μL mL^–1^ antibiotic cocktail (P4083; Merck) and incubated under dark conditions at 19–20°C. Cultures of FC901 and SRM-001 were established from the incubated samples by isolating a single cell using a glass micropipette, and cultures were axenically maintained by an inoculation in Hemi medium without antibiotics at 19–20°C under dark conditions every 2‍ ‍weeks. The absence of contaminating bacterial cells in the culture (*i.e.*, an axenic culture) was confirmed by careful microscopic observations and PCR of total DNA using the universal bacterial primer set 27f (Lane, 1991) and 1492r (Edwards *et al.*, 1989). Living FC901 and SRM-001 cells were observed using a BX43 microscope (Olympus) equipped with the digital 4K camera, FLOYD-4K (Wraymer).

### Sequencing ana­lyses

Approximately 200‍ ‍mL from cultures of FC901 and SRM-001 at the mid-exponential phase was centrifuged at 2,400×*g* for 5‍ ‍min. Cell pellets were frozen and sent for sequencing (Azenta). DNA was extracted from cell pellets using an Invitrogen PureLink Genomic DNA Mini Kit (Thermo Fisher Scientific), and libraries were constructed using a VAHTS Universal Pro DNA Library Prep Kit for Illumina (Vazyme) for 2×150 bp PE sequencing using NovaSeq 6000. Total RNA was extracted from cell pellets using a Qiagen RNeasy Mini Kit (Qiagen) or TRI Reagent (Thermo Fisher Scientific) and cleaned using an RNA Clean & Concentrator Kit (Zymo Research). The enrichment and purification of mRNA were conducted using an NEBNext Poly(A) mRNA Magnetic Isolation Module (New England Biolabs), and libraries were constructed using an NEBNext Ultra II Directional RNA Library Prep Kit for Illumina (New England Biolabs) for 2×150 bp PE sequencing by NovaSeq 6000 or MGIEasy RNA Directional Library Prep Set v.2.0 (MGI) for 2×150 bp PE sequencing using DNBSEQ-G400. The kits and sequencing platforms used for each ana­lysis are summarized in Table S2.

Raw FASTQ data from genomic DNA sequencing (DNA-seq) was quality-trimmed, and the adapter sequences were removed using Atria v. 4.0.0 (Chuan *et al.*, 2021) with a default setting (*i.e.*, average quality threshold: 20, sliding window length: 5, fastq quality format 33). Filtered reads were divided into 100 subsets using SeqKit v. 2.1.0 (Shen *et al.*, 2016), and three subsets of each strain were subjected to contig assembly using SPAdes v.3.13 (Bankevich *et al.*, 2012). A single possible mitochondrial genomic fragment from each assembly contig dataset was detected by BLASTN using the mitochondrial genome sequence of *Ophirina amphinema* (GenBank accession number: LC369600.1) as the query sequence. The detected sequences were identical among the three subset ana­lyses of each strain; however, the starting position of each sequence differed. To confirm the existence or absence of DNA heterogeneity among the chromosomes, filtered raw reads were mapped onto the reconstructed genome of each strain using HISAT2 v. 2.2.1 (Kim *et al.*, 2019), and heterogeneity at each site was calculated using REDItools 2.0 (Flati *et al.*, 2020).

The raw FASTQ data of transcriptional data sequencing (RNA-seq) were also quality-trimmed, and the adapter sequences were removed using Atria v. 4.0.0 with a default setting. An RNA-seq ana­lysis of FC901 was conducted three times, and data were combined into a single dataset and analyzed (Table S2). Filtered raw reads were mapped onto the reconstructed genome of each strain using HISAT2 v. 2.2.1. The presence and level of RNA editing at each site were detected using REDItools 2.0. The sites mapped by more than 10 filtered reads were counted and subjected to further ana­lyses. The level of RNA editing at each site was calculated using the following formula (Chigaev *et al.*, 2019):

RNA editing level

$$=\frac{\mathrm{number of reads supporting the edited allele}\times 100}{\mathrm{total number of reads at a site}}$$

Gene annotation in mitochondrial genomes was conducted with sequences that were mediated at the more than 50% RNA editing level using MFannot (https://megasun.bch.umontreal.ca/apps/mfannot/). Editing sites in protein-coding regions were analyzed to calculate the ratio of nonsynonymous to synonymous substitutions using KaKs_calculator v.3.0 (Zhang, 2022) with the NG method. Codon positions in each gene were identified using Mesquite v.3.10 (Maddison and Maddison, 2011), and the editing level at each site was visualized as a heatmap using Microsoft Excel.

The filtered RNA-seq reads of each strain were subjected to contig assembly using Trinity v.r2012-10-05 (Grabherr *et al.*, 2011). We searched the assembled contigs for transcripts of PPR-DYW protein by TBLASTN using a previously used query sequence (Schallenberg-Rüdinger *et al.*, 2013). We searched for transcripts of proteins with PPR motifs using the same approach as the corresponding gene sequences of *Acanthamoeba castellanii* (ELR25026.1), *Ectocarpus siliculosus* (CBN80012.1), and *Klebsormidium nitens* (GAQ77902.1). The E-value cut-off was set to 10^–3^ in both surveys. The detected sequences were then subjected to TPRpred (Karpenahalli *et al.*, 2007) and identified as PPR proteins. The fungal sequences of PPR-DYW proteins were also searched from the GenBank nucleotide collection database using the same approach as above. The DYW domains in the detected sequences were manually aligned with previously reported sequences (Schallenberg-Rüdinger *et al.*, 2013), and the PPR motifs were profiled using WebLogo (Crooks *et al.*, 2004). Prior to a phylogenetic ana­lysis, the aligned DYW domain sequences were prepared by removing unique insertions found in less than five taxa and partial sequences (*e.g.*, SRM-001c). The tree topology and branch lengths were inferred through the maximum likelihood (ML) method using IQ-TREE v.2.2.0 (Nguyen *et al.*, 2015) with the LG+I+G4 model that was selected as the best fit model in the “-m TEST” option. A non-parametric bootstrap ana­lysis was performed with 100 replicates under the LG+ I+G4 model. A Bayesian phylogenetic ana­lysis was conducted with the CAT+GTR model using PhyloBayes v.2.1 (Lartillot and Philippe, 2004; Lartillot *et al.*, 2013), which included two Markov chain Monte Carlo runs of 100,000 cycles with a “burn-in” of 25,000 cycles that sufficiently converged at a maxdiff score of 0.029. The subcellular localization of detected PPR proteins was analyzed by MitoFates v.1.2 (https://mitf.cbrc.pj.aist.go.jp/MitoFates/cgi-bin/top.cgi) (Fukasawa *et al.*, 2015) with “fungi” selected in the organism option; the “metazoa” and “plant” options were also selected and analyzed, and the results obtained were identical. In the search of the RNA-seq data‍ ‍of FC901 and SRM-001 for candidates of ADAR-like and ADAT sequences, the ADAR-like sequence of *Symbiodinium microadriaticum* was used as a query (OLQ07757; the E-value cut-off was set to 10^–10^). We also searched publicly available sequencing data for the ADAR-like and ADAT sequences of other protists using the same approach. The detected sequences (*e.g.*, ADAR-like and ADAT of *Phaeodactylum tricornutum*) were also used as queries in a further search to identify additional ADAR-likes and ADATs. The ADAR-like sequences detected in protist species were aligned with the metazoan ADARs and ADATs using MAFFT v.7.471 with the “L-INS-i” option (Katoh *et al.*, 2002, 2014). The aligned sequences were masked for a phylogenetic ana­lysis using trimAl v.1.4 with the “strict” option (Capella-Gutiérrez *et al.*, 2009). This initial dataset contained all detected sequences, including partial and/or highly divergent sequences, and only 94 positions were included in the phylogenetic ana­lysis. The tree topology and branch lengths were inferred using IQ-TREE v.2.2.0 as stated above, and the main dataset was prepared by excluding 14 partial and divergent ADAR-like sequences in the initial alignment. The main dataset was analyzed using the same methods as those stated above: the LG+F+I+G4 model was selected as the best-fit model and subjected to the ML ana­lysis, and convergence in the Bayesian ana­lysis was confirmed by the maxdiff score (0.076) with a “burn-in” of 25,000 cycles in 100,000 cycles. The 13 ADAR-like sequences retained in the main dataset were subjected to motif identification by HMMER v.3.3 (http://hmmer.org) against the Pfam database (Mistry *et al.*, 2021). The mole­cular weight of ADAR-like from FC901 was estimated from the amino acid sequence using the Peptide and Protein Molecular Weight Calculator (https://www.aatbio.com/tools/calculate-peptide-and-protein-mole­cular-weight-mw). The subcellular localization of ADAR-like from FC901 was predicted using MitoFates v.1.2 with the “fungi” option. The subcellular localization of ADAR-likes from SRM-001 was not analyzed because the 5′-end region was not completely sequenced. The assembled contigs were also subjected to a BUSCO v.5.0.0 ana­lysis (Manni *et al.*, 2021) to assess the volume of RNA-seq with the “-m transcriptome” and “--auto-lineage-euk” options.

The 18S rRNA gene sequences of FC901 and SRM-001 were elu­cidated using DNA extracted from a 20-mL culture with a Qiagen DNeasy Plant Mini Kit (Qiagen) using the Euk1A (Sogin and Gunderson, 1987) and EukB (Medlin *et al.*, 1988) primers. Sequences were added to the alignment generated as previously described (Ward *et al.*, 2018) and aligned using MAFFT v.7.471 with a default setting. The 18S rRNA gene sequences of some ascetosporeans, such as mikrocytids, were too divergent to be included and aligned. The ML tree with non-parametric bootstrap ana­lyses of 1,000 replicates and the Bayesian tree were reconstructed as previously described (Yabuki *et al.*, 2023).

### Immunofluorescence ana­lysis

Cells of FC901 and SRM-001 were fixed with 4% paraformaldehyde in cultivation medium, centrifuged at 2,400×*g* for 5‍ ‍min, and then embedded in 1% agarose in 0.22-μm filtered artificial seawater (FASW, 3.5% Rei-Sea Marine II; Iwaki). Cells in the agarose gel were washed with FASW, dehydrated in a graded series of ethanol (30%, 50%, 70%, 90%, and 100%), and embedded in Technovit 8100 resin (Mitsui Chemicals) at 4°C. Semi-thin sections (thickness of approximately 1‍ ‍μm) were cut using a glass knife mounted on an Ultracut S ultra-microtome (Danaher) and collected on a glass slide. Sections were treated with 2% block ace (KAC) in 1× phosphate-buffered saline (PBS) at room temperature for 20‍ ‍min and then incubated with an anti-ADAR antibody (HPA051519; Merck) diluted 1:200 in PBS at 37°C for 12 h. Sections were then incubated with a CF565-conjugated goat anti-rabbit IgG secondary antibody (1:200 dilution in PBS; Nacalai Tesque) at room temperature for 2‍ ‍h and stained with 1‍ ‍μM Mito View Green solution (Biotium) and 4′,6-diamidino-2-phenylindole (DAPI) for 30 and 5‍ ‍min, respectively. Sections were observed using a BX-51 light and fluorescence microscope (Olympus) with UV (excitation, 330–385‍ ‍nm; emission, >400‍ ‍nm), FITC (excitation, 470–495‍ ‍nm; emission, 510–550‍ ‍nm), and CY3 (excitation, 530–570‍ ‍nm; emission, 573–648‍ ‍nm) filter sets for DAPI, antibodies, and Mito View Green, respectively.

## Results and Discussion

### Paradinid culture, mitochondrial genome, and RNA editing

**Fig. 1.**
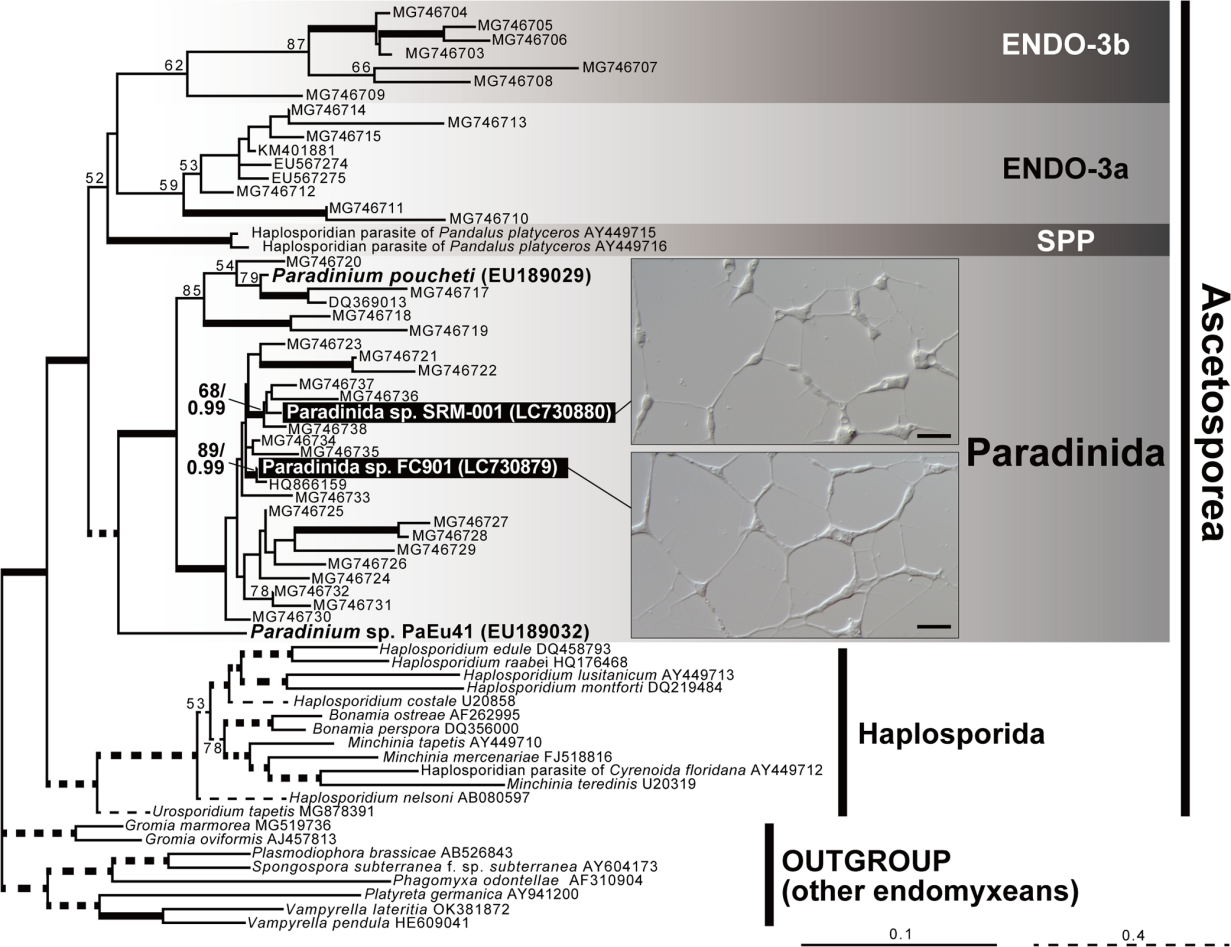
Phylogenetic tree of endomyxean 18S rRNA gene sequences with light microscopic images of Paradinida spp. FC901 and SRM-001. The analyzed dataset contained 67 OTUs with 1,856 nucleotide positions and the TN+F+I+G4 model was subjected to a ML ana­lysis. Only ML bootstrap values >50% are shown. The branches supported by both >90% ML bootstrap value and >0.95 Bayesian posterior probability are shown in bold lines. Scale bar, 20‍ ‍μm.

Two ascetosporean strains, FC901 and SRM-001 (Fig. 1, S1, and S2), were established as clonal and axenic cultures. They phylogenetically belong to Paradinida (paradinids), Ascetosporea, Rhizaria in the 18S rRNA gene tree with the highest statistical support (Fig. 1). All species belonging to Ascetosporea are parasitic organisms; therefore, FC901 and SRM-001 may also exhibit parasitism in natural environments, similar to other ascetosporean species. However, the diversity of Ascetosporea has yet to be exami­ned in detail, and the existence of some free-living and/or amphizoic members cannot be ruled out. In the present study, we tentatively treated them as unidentified species belonging to the order Paradinida, *i.e.*, Paradinida spp., based on their phylogenetic affiliation; however, further studies are needed to elucidate their proper taxonomic identification (see Supplementary Information for a detailed taxonomic discussion).

**Fig. 2.**
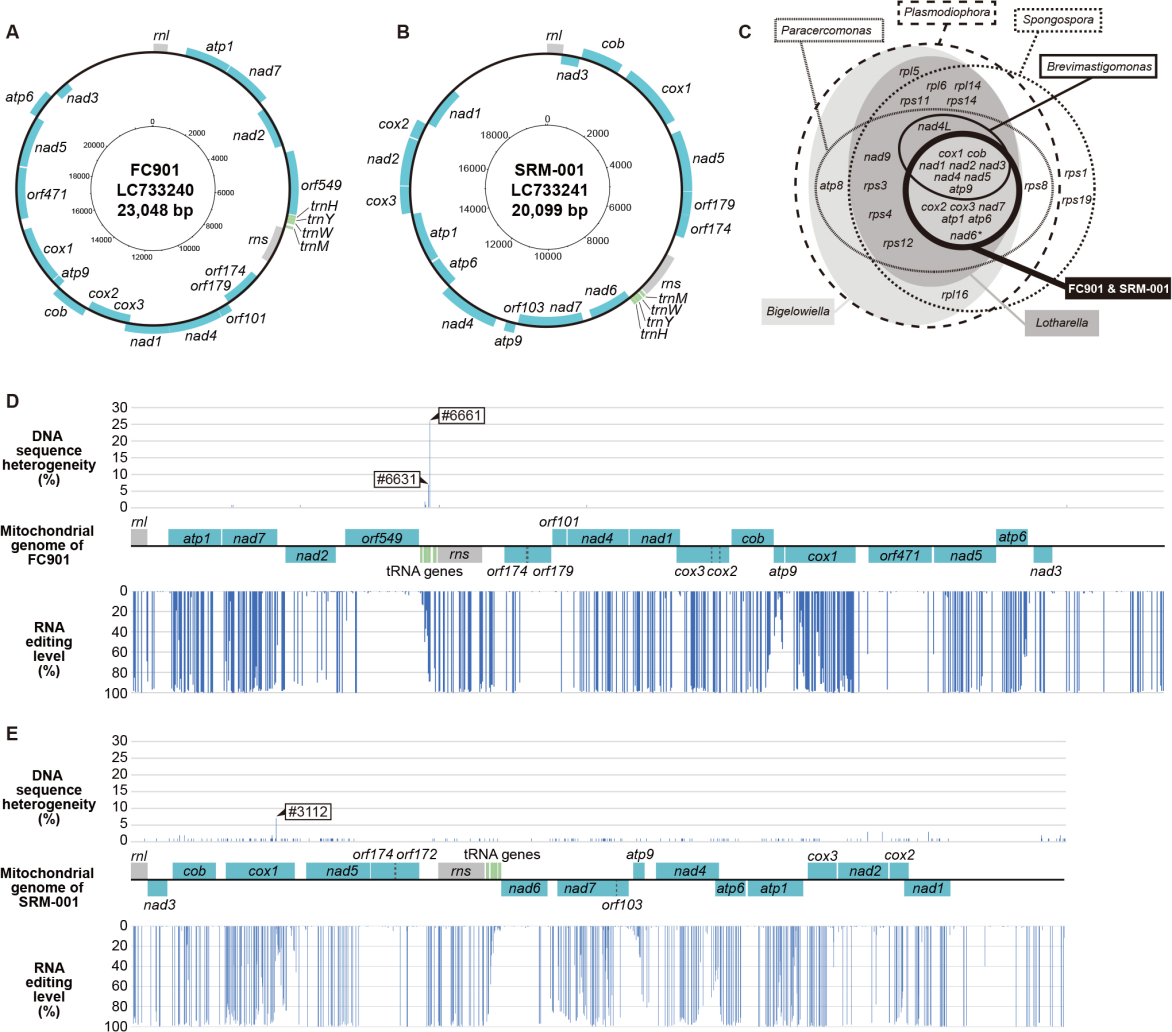
Summary of mitochondrial genomes of FC901 and SRM-001 **A.** **B.** Mitochondrial genome maps of Paradinida spp. FC901(A) and SRM-001 (B). Protein-coding regions are shown in pale blue. Transfer RNA genes and other structural RNA genes (*rns* and *rnl*) are shown in green and gray, respectively. **C. A** Venn diagram summarizing protein-coding genes on the mitochondrial genomes of related rhizarians. Note that *nad6* is absent in the mitochondrial genome of FC901, but present in that of SRM-001. **D, E.** Mitochondrial genomes of FC901 (D) and SRM-001 (E) with information regarding heterogeneity in raw DNA-seq reads and editing levels calculated from raw RNA-seq reads.

Circularly mapped mitochondrial genomes with lengths of 23,048 and 20,099 bp in FC901 (LC733240) and SRM-001 (LC733241), respectively, were reconstructed (Fig. 2A and 2B). Since the reliability of the reconstructed mitochondrial genomes was confirmed by mapping of the filtered DNA-seq reads, only two sites in FC901 (#6661 with 26.6% and #6631 with 7.3%) and one site in SRM-001 (#3112 with 7.0%) were detected with DNA heterogeneity more than 5%, with a score of zero being observed at most sites (Fig. 2D and 2E, Table S3). DNA-seq reads were well mapped onto the reconstructed genomes (Fig. S3A and S3B), and the average number of the reads covering each site was 9,862 for FC901 and 1,894 for SRM-001. Therefore, we considered the reconstructed mitochondrial genomes deposited as LC733240 and LC733241 to generally be reliable, although a few minor variations in mitochondrial genomes corresponding to the detected DNA heterogeneity may exist.

The coding regions in their genomes were fragmented by many unexpected in-frame stop codons, suggesting that they were either pseudogenes or involved in RNA editing. To confirm these possibilities, we compared them with RNA-seq data, which revealed that there were nucleotide mismatches between DNA-seq and RNA-seq data at many sites (Fig. 2D and 2E). In more detail, 338 adenosine (A) and 20 cytidine (C) residues in FC901 and 306 A and 21 C residues in SRM-001 were switched to guanosine (G) and uridine (U) in RNA-seq data at the 50% editing level threshold (Table S4). Since the adenosine-to-inosine (I) substitution is the most common type of RNA editing, and inosine is recognized as guanosine in reverse transcription in addition to the detection of ADAR-like sequences from two paradinids (discussed below in more detail), we considered the paradinids to also possess A-to-I substitutions rather than A-to-G substitutions, in addition to the C-to-U substitution. Although the C-to-U substitution has been reported in the mitochondria of various eukaryotes, such as plants, heteroloboseans, dinoflagellates, and diplonemids (Lin *et al.*, 2002, 2008; Fu *et al.*, 2014; Moreira *et al.*, 2016; Small *et al.*, 2020; Knoop, 2023), it has never been reported in rhizarian protists. Mitochondrial A-to-I substitutions have only been found in diplonemids to date (Moreira *et al.*, 2016), while A-to-G substitutions have been reported in the mitochondria of dinoflagellates (Lin *et al.*, 2002; Lin *et al.*, 2008), and the possibility that they are A-to-I substitutions in the natural state still remains. Since mitochondrial C-to-U and A-to-I(G) substitutions have rarely been reported and paradinids are phylogenetically distinct from diplonemids and dinoflagellates, it is reasonable to speculate that their mitochondrial RNA editing evolved independently. Other types of RNA editing, such as another type of substitution and nucleotide insertion, were not observed in the present study.

**Fig. 3.**
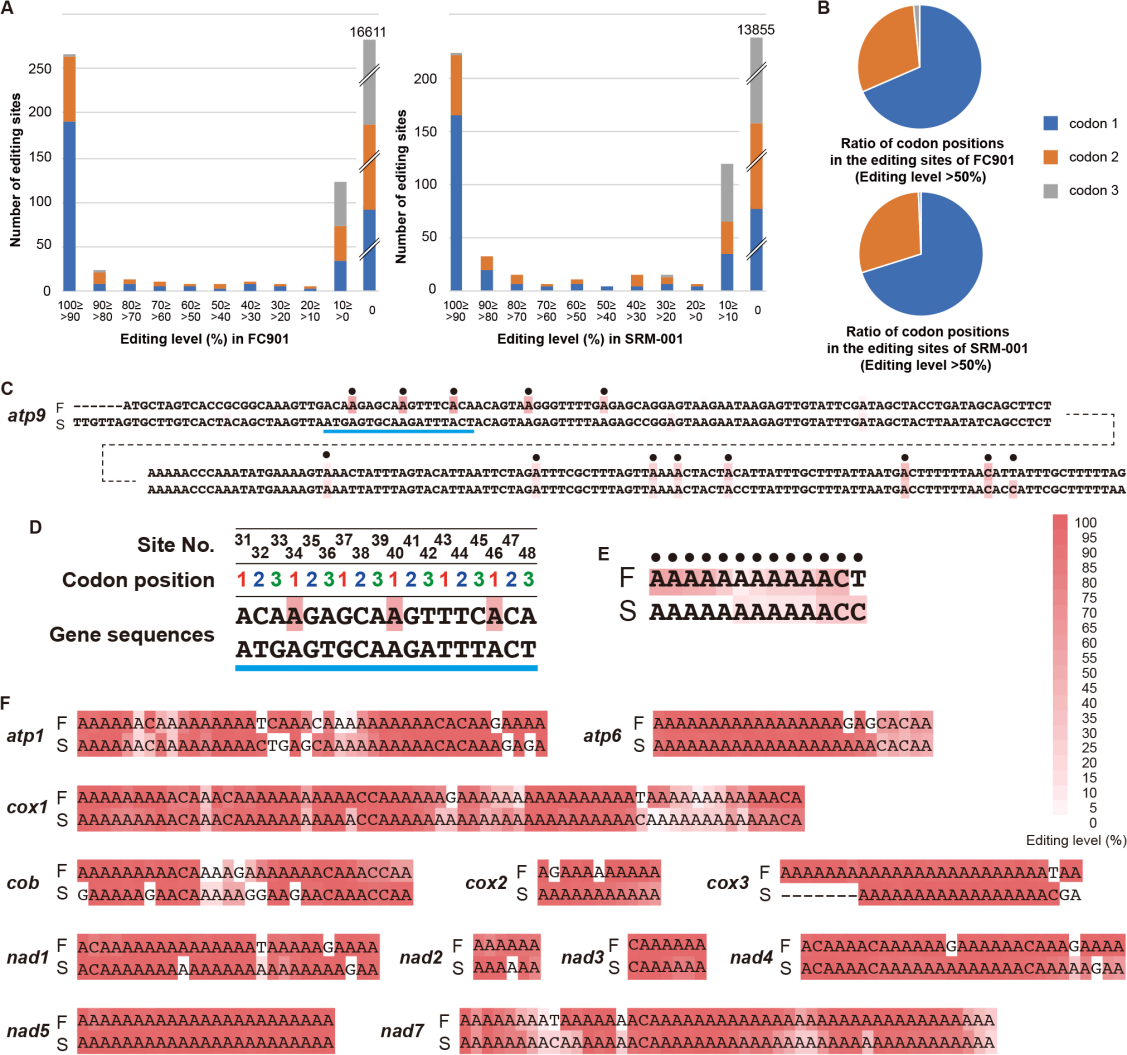
Summary of RNA editing in two paradinids. **A.** Bar graph summarizing the number of sites per editing level in FC901 (left) and SRM-001 (right). **B.** Pie chart summarizing the composition of codon positions detected at the more than 50% editing level in FC901 (upper) and SRM-001 (lower). **C.** DNA sequences of *atp9* in FC901 (F) and SRM-001 (S) with information on the editing level (red heatmap). **D.** Enlarged view of the DNA regions indicated by blue underlines in 3C with information regarding site numbers and codon positions. **E.** Summary of the editing sites and level of *atp9*. The editing sites detected at the more than 10% level in either FC901 or SRM-001 (indicated by black dots in 3C) were extracted and lined. **F.** Summary of the editing sites of the other protein-coding genes as shown under the same rule of *atp9* (3E).

Editing sites were broadly distributed within the mitochondrial genomes of two paradinids, and all genes, except for a few tRNA genes and unannotated reading frames, were involved in RNA editing (Fig. 2D and 2E, Table S4). Density varied gene by gene, and approximately 2% of the‍ ‍mitochondrial genome sequences were affected by RNA‍ ‍editing in paradinids (Table S4). This is similar to those of other protists possessing organellar RNA editing (Bundschuh *et al.*, 2011; Grewe *et al.*, 2011; Oldenkott *et al.*, 2014; Klinger *et al.*, 2018), but markedly lower than the highest rate (12.2%) of the diplonemid *Namystynia karyoxenos* (Kaur *et al.*, 2020). Most sites (84.4 and 77.4% of editing sites of the protein-coding regions in FC901 and SRM-001, respectively) were edited at the more than 90% editing level (Fig. 2D, 2E, and 3A, Table S5), suggesting that substitutions at these sites progressed promptly after transcription. The editing level in paradinid mitochondria is similar to that in plants because approximately 15% of editing sites in plant mitochondria were partially substituted at the 90% editing level or less (Mower and Palmer, 2006; Picardi *et al.*, 2010; Grewe *et al.*, 2011). In contrast, sites with low editing levels were also present and dominant in the transcripts of *atp9* (Fig. 3C, 3D, and 3E, Table S6). The transcriptional level of *nad2* was as low as that of *atp9* (Fig. S3A and S3B, Table S6); however, the editing level of *nad2* was markedly higher than that of *atp9* (Fig. S3C, Table S6) and the relationship between editing and transcription levels was not significant (Table S6). It currently remains unclear why and how editing levels were controlled in different manners. Many editing sites were shared between FC901 and SRM-001 (Fig. 3E and 3F), indicating that some editing sites existed in a common ancestor. Nevertheless, several editing sites unique to strains also existed (Fig. 3E and 3F), indicating that the acquisition of additional editing sites is currently progressing or has recently evolved. The distribution of editing sites was primarily at the first and second codon positions (Fig. 3B), and all edits were nonsynonymous substitutions (Table 1). This ratio of nonsynonymous RNA editing has not yet been reported in any taxa. In plants, the majority of RNA-editing events also occur at the first and second positions of codons, yet only 90% of edits are nonsynonymous codon changes (Yura and Go, 2008; Edera *et al.*, 2018). The ratios of nonsynonymous RNA editing in diplonemids and dinoflagellates were similarly high in some species (Lin *et al.*, 2008; Moreira *et al.*, 2016), but did not account for 100% of mitochondrial substitutions. These findings suggest more stringent evolutionary constraint for RNA editing at these codon positions than for other RNA editing phenomena. In addition, most substitutions in paradinids contributed to changes in the physicochemical properties of amino acids (Fig. S4). When editing occurred within codon frames corresponding to well-conserved amino acids, the ancestral amino acid was restored (Fig. S5). Therefore, RNA editing in paradinids may restore RNA sequences to keep functional amino acid sequences, as discussed in previous studies on organellar RNA editing (Chateigner-Boutin and Small, 2011; Lukes *et al.*, 2021).

Although complete mitochondrial genomes were not available for ascetosporeans until the present study, a fragment was reported from the haplosporidian *B. ostreae* (Hiltunen Thorén *et al.*, 2024). All protein-coding genes in the mitochondrial genome of *B. ostreae* were also detected in those of paradinids; however, *trnI*, *trnV*, and *trnQ* were unique in *B. ostreae*. Only four species of tRNA genes were annotated in the mitochondrial genomes of paradinids, while other tRNA species may be encoded on their nuclear genomes and transported into the mitochondria, as reported in other protists (Gray *et al.*, 1998; Tan *et al.*, 2002; Kolesnikova *et al.*, 2004; Lang, 2018). Protein-coding genes in the mitochondrial genomes of paradinids were involved in the electron transport chain (Fig. 2C), suggesting that ATP is synthesized via the electron transport chain within the mitochondria of paradinids, unlike MROs in Mikrocytida. Since *B. ostreae* also possesses some of the same protein-coding genes, some haplosporidians may also synthesize ATP via the electron transport chain in their mitochondria. Several well-conserved residues were substituted in the COX1 and COB amino acid sequences of *B. ostreae* that were translated from genomic data (Fig. S5), suggesting that RNA editing also occurred in the mitochondria of *B. ostreae*; however, this needs to be confirmed in future studies. The function of ascetosporean mitochondria, including MROs, may differ lineage-by-lineage based on the organisms they parasitize. Paradinida species are known to parasitize the body caves and intestines of small copepods (Shields, 1994; Skovgaard and Daugbjerg, 2008), and these environments may be more aerobic than those that target the interior of organismal tissue. Since mole­cular oxygen may be utilized there, their mitochondria may still be retained.

### Detection of PPR-DYW protein and ADAR-like in paradinids and their origins

**Fig. 4.**
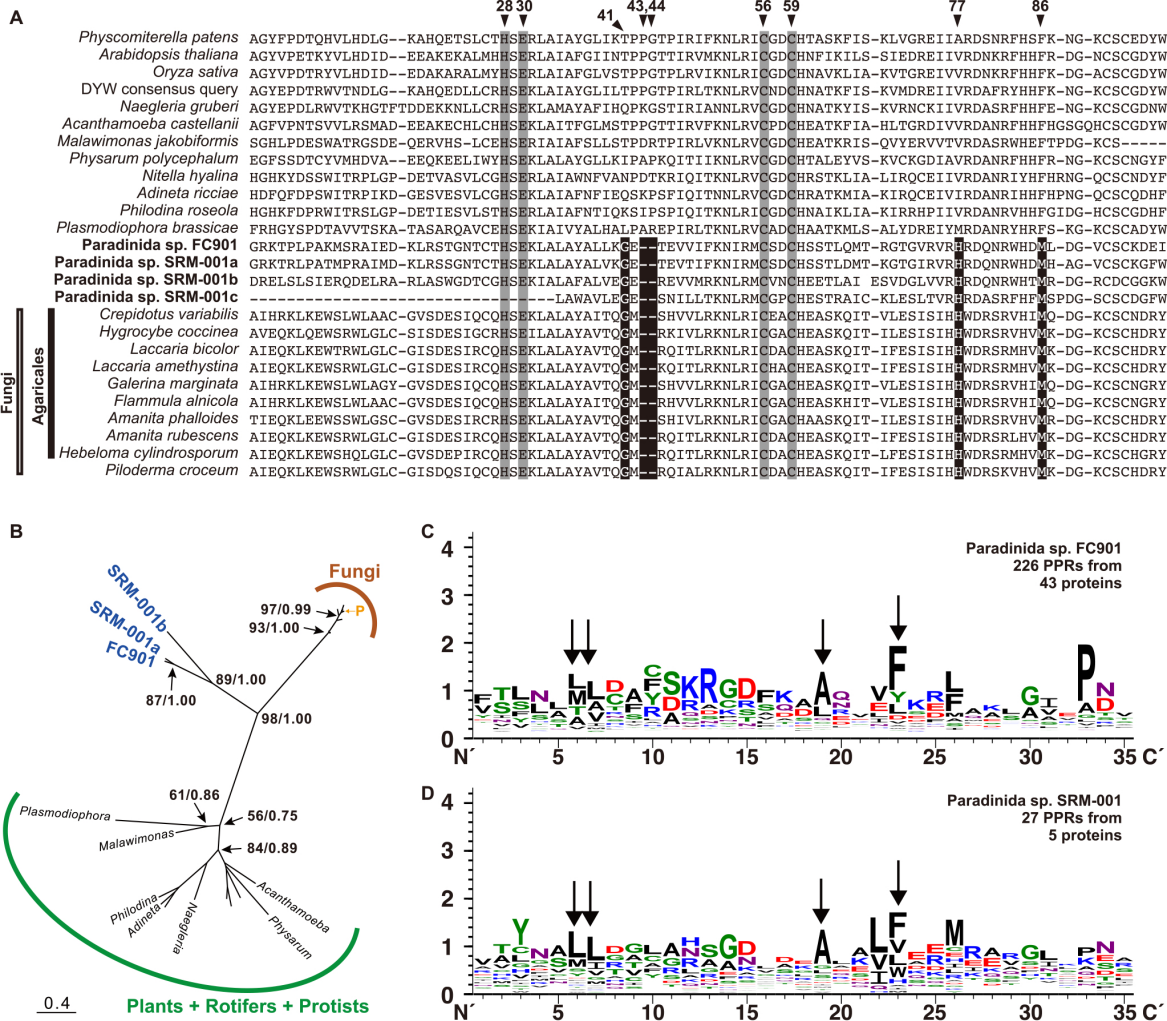
Summary of PPR protein ana­lyses. **A.** Alignments of DYW domains. Highly conserved amino acids are shown on a gray background. Unique deletion and specific amino acids that are only shared by fungi and paradinids are highlighted in white text on a black background. **B.** The unrooted ML tree inferred from DYW domains. The analyzed dataset contained 24 OTUs with 92 amino acid positions and LG+I+G4 was subjected to an ML ana­lysis. Bootstrap values and Bayesian posterior probabilities are shown only at major selected branches. Fungal and plant sequences are shown as branches omitting species names, and “P” in the clade of fungi indicates *Piloderma croceum*. **C. D.** Weblogo profiles of PPR motifs found in PPR-DYW and P-type PPR proteins in FC901 (C) and SRM-001 (D). Black arrows indicate the positions where the same amino acid is conserved in two paradinids.

PPR-DYW protein was originally identified as a key enzyme for C-to-U substitutions in plant mitochondria and has been reported in plant chloroplasts (Takenaka *et al.*, 2013). A few protists also possess this enzyme, and C-to-U substitutions similarly occur within their mitochondria (Knoop and Rüdinger, 2010; Schallenberg-Rüdinger *et al.*, 2013). We found one sequence in FC901 and three sequences in SRM-001 for PPR-DYW protein (Fig. 4A), indicating that the use of PPR-DYW is more widespread than previously known. Furthermore, we detected PPR-DYW protein sequences from several fungal species, although it has only been previously recorded in one species, *Laccaria bicolor* (Lurin *et al.*, 2004; Schallenberg-Rüdinger *et al.*, 2013). Well-conserved residues, including histidine at the 28^th^ position, glutamate at the 30^th^ position, and cysteines at the 56^th^ and 59^th^ positions in the alignment (Schallenberg-Rüdinger *et al.*, 2013) were also consistently shared in the PPR-DYW protein sequences of paradinids and fungi (Fig. 4A); therefore, their sequences may be regarded as PPR-DYW proteins. This was also supported by the predicted localization of PPR-DYW protein in the mitochondria of FC901 and seven fungi (Table S7), while PPR-DYW protein from SRM-001 was only partially obtained and not subjected to the prediction by MitoFates. The PPR-DYW protein sequences of paradinids and fungi shared a unique deletion at the 43^rd^ and 44^th^ positions in the alignment and conserved amino acids at several positions (Fig. 4A), suggesting that their sequences share the same origin. Molecular phylogeny also supported this speculation (Fig. 4B), and the ancestral form of their PPR-DYW proteins may have been obtained from a plant species via HGT; however, further studies are needed to obtain a more detailed understanding of this process (*e.g.*, the first recipient and direction of HGT in paradinids/fungal clade). Moreover, all fungal members possessing PPR-DYW protein belonged to the order Agaricales, except for *Piloderma croceum* (order Atheliales), which branched within the clade of Agaricales with high statistical support (Fig. 4B). The PPR-DYW protein of *P. croceum* may have been obtained from a species of Agaricales via HGT; however, this requires further study of their mitochondrial C-to-U substitutions.

In addition to PPR-DYW proteins, we detected P-type PPR proteins that did not possess DYW from FC901 or SRM-001 (42 from FC901 and two from SRM-001) (Fig. 4C and 4D). Since P-type PPR proteins are involved in organellar RNA editing (Takenaka *et al.*, 2013), some may play a role in the mitochondrial RNA editing of paradinids. Each C-to-U editing site is specifically recognized by its corresponding PPR motif in plant organelles (Okuda and Shikanai, 2012), and FC901 possesses more than 20 C-to-U editing sites (Table S4). In the present study, 12 of the 42 P-type PPR proteins of FC901 were predicted for mitochondrial localization (Table S7), and some may be involved in the C-to-U substitution. If the mediating mechanism in paradinids is similar to that in plants, several other mitochondrial-targeting P-type PPR proteins may be present. Some P-type PPR proteins that were not predicted may be targeted to the mitochondria and may be able to recognize more than two editing sites in paradinids. BUSCO scores in the RNA-seq ana­lysis were 85.1% (FC901) and 62.7% (SRM-001) (Table S2); therefore, a full set of P-type PPR proteins may not have been sequenced, with only two P-type PPR proteins being detected from SRM-001. Several amino acid residues, such as leucine (L) at the 6^th^ and 7^th^ positions, alanine (A) at the 19^th^ position, and phenylalanine (F) at the 23^rd^ position were conserved in their PPR motifs (Fig. 4C and 4D). Some were also conserved in the PPR motifs of other protist species (Schallenberg-Rüdinger *et al.*, 2013) and may be involved in important functions that have not yet been exami­ned in detail.

**Fig. 5.**
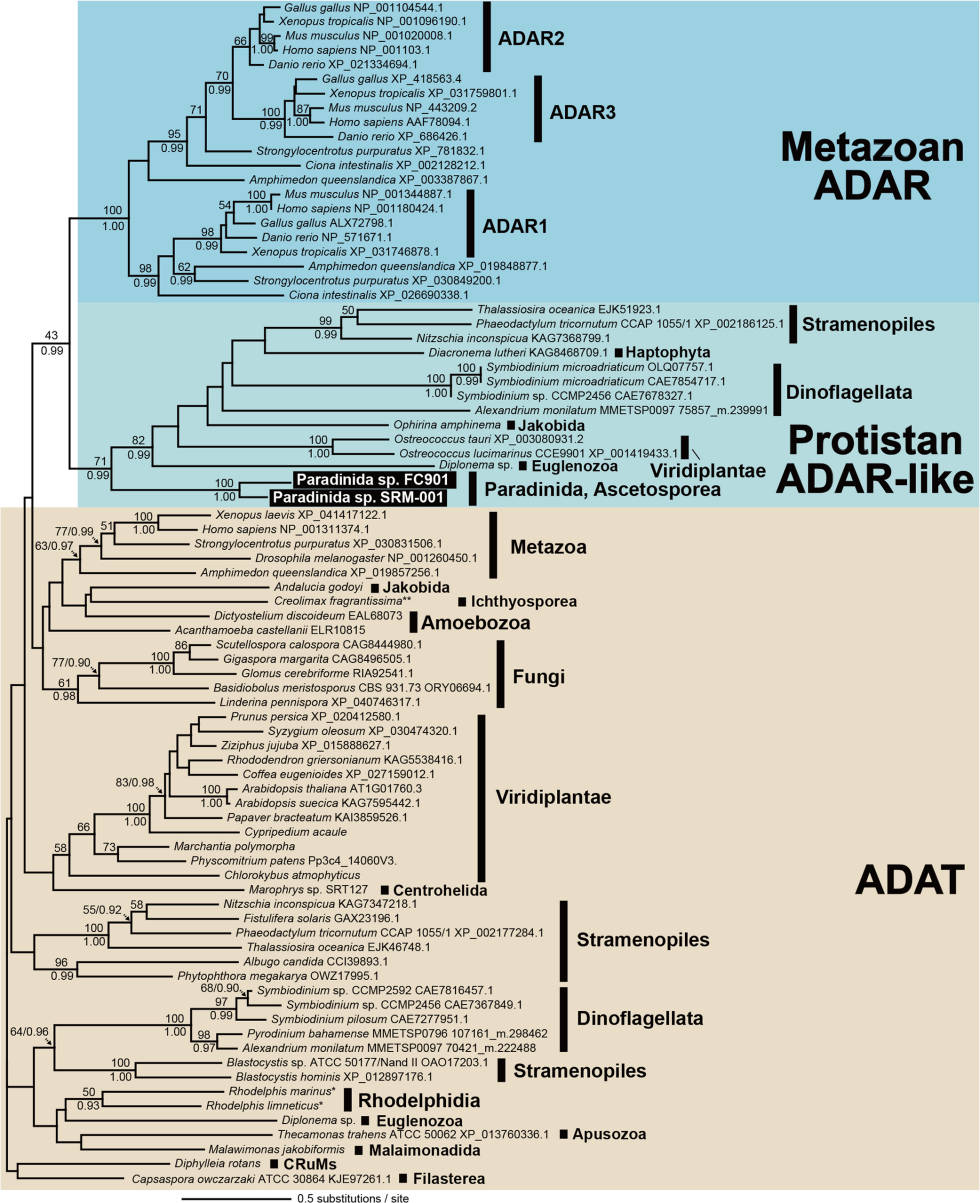
Phylogenetic tree of eukaryotic ADAR and ADAR-like sequences rooted with eukaryotic ADAT. ADAT, metazoan ADARs, and protistan ADAR-like sequences are shown on brown, blue, and light green backgrounds, respectively. The analyzed dataset contained 211 OTUs with 96 amino acid positions and the LG+F+I+G4 model was subjected to an ML ana­lysis. Only ML bootstrap values/Bayesian posterior probabilities ≥50%/0.90 are shown.

The detection of ADAR-like in paradinids is an important result because it has only been previously detected in *Symbiodinium* spp. (Fig. 5). Furthermore, when we searched publicly available sequencing data for ADAR-like (Table S8), 28 ADAR-like sequences from 24 protist species were identified. Since 14 of the 28 sequences were partial and/or highly divergent, their assignment is not yet conclusive. The remaining 14 sequences from 13 protists, including two paradinids, formed a clade with moderate statistical support, while the sister relationship between protistan ADAR-likes and metazoan ADARs was not well supported (Fig. 5). Additionally, ADAT was found in 9 of the 13 protist species that possessed ADAR-like (Table S8). The double-strand RNA-binding domain, a key domain of metazoan ADARs, was absent in protistan ADAR-likes; however, several other domains were detected (Fig. S6). Protists possessing ADAR-like belong to a phylogenetically divergent lineage yet share the same origin with metazoan ADARs; therefore, although the last eukaryotic common ancestor (LECA) may have had its ancestral form and was vertically inherited through eukaryotic diversification, HGT of ADAR-like among protists may also have occurred. This result shows that the origin of metazoan ADAR may have evolved much earlier than previously considered, and the secondary loss of ADAR or ADAR-like may have occurred independently at the base of fungi, which do not have ADAR (Teiche, 2020).

**Fig. 6.**
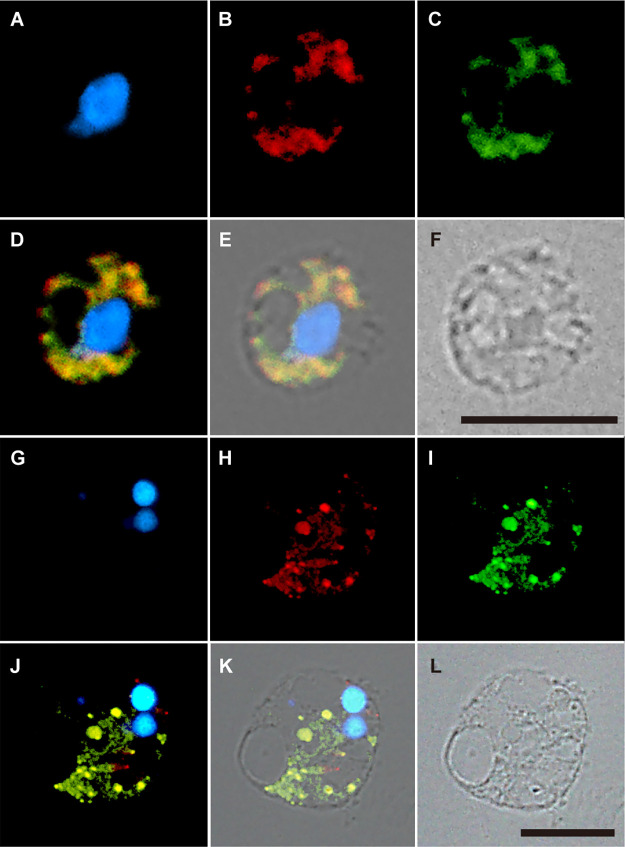
Subcellular localization of ADAR-like in paradinids. **A, B, C, D, E, and F**: FC901, **G, H, I, J, K, and L**: SRM-001. Cell sections were stained with DAPI (**A, G**), an anti-ADAR antibody (**B, H**), and Mito View (**C, I**). Differential interference contrast images of cell sections (**F, L**). **D.** Merged view of A, B, and C. **E**. Merged view of A, B, and C and F. **J.** Merged view of G, H, and I. **K.** Merged view of G, H, and I and L. Bar, 10‍ ‍μm.

Although ADAR-likes have been detected in paradinids, whether these proteins mediate the A-to-I substitution in mitochondria has not been directly demonstrated. All ADARs exami­ned to date are from metazoans and localize within the nucleus, except for a single isoform found in the cytosol (Ashley *et al.*, 2024). ADAR-like in FC901 was not predicted to be localized within mitochondria by Mitofates (Table S7), whereas ADAR-likes in SRM-001 were partially obtained and, thus, were not analyzed by Mitofates. In contrast, the structures stained by the anti-human-ADAR antibody clearly overlapped with mitochondria stained by Mito View (Fig. 6) from both paradinids. These results support the hypothesis that ADAR-like is targeted to mitochondria and mediates the A-to-I substitution in paradinids. The antigenic site of this protein is localized in the deaminase domain, which was highly conserved in all ADARs and protistan ADAR-likes (Fig. S6). While a single and clear signal was detected in the Western blot ana­lysis, the protein size (50‍ ‍kDa) was markedly smaller than the estimated size (187‍ ‍kDa) of ADAR-like in FC901 (Fig. S7). Since no proteins in FC901, except for ADAR-like, possessed a deaminase domain similar to human ADAR, it is possible that more than two-thirds of the 5′ region of mRNA was not translated. Although its localization within mitochondria was not predicted by MateFates (Table S7), if the 10^th^ methionine was a start codon, the protein size was estimated to be 53‍ ‍kDa (Fig. S6). Another possibility is that the N-terminal re­gion of ADAR-like was truncated during the transport pro­cess, although only one signal was detected by the Western blot ana­lysis (Fig. S7). The results of immunostaining were clear and those of the MitoFates ana­lysis did not deny the subcellular localization of ADAR-like in mitochondria; there­fore, we currently consider the ADAR-like of paradinids to be involved in mitochondrial RNA editing, which needs to be carefully confirmed in future research.

The subcellular localization of ADAR-likes in other protist species was not analyzed in the present study. Among the protists possessing ADAR-like, only dinoflagellates, diplonemids, and paradinids possess the A-to-I(G) substitution in their mitochondrial genes (Lin *et al.*, 2008; Moreira *et al.*, 2016, the present study). Although the mitochondrial genomes of other protists have already been sequenced and reported (Secq and Green, 2011; Hulatt *et al.*, 2020; Oliver *et al.*, 2021), the possible existence of A-to-I substitutions in their mitochondria has not been confirmed. Therefore, further studies are needed to clarify the sites at which and the mechanisms by which their ADAR-likes function.

## Conclusion

The present study uncovered the mitochondrial genomes of ascetosporean paradinids, a poorly understood marine parasite, and exami­ned RNA editing occurring in their mitochondria. Functional differences in ascetosporean mitochondria/MROs may depend on their adaptation to anaerobiosis for a parasitic lifestyle; however, the complete life history of any ascetosporean has not yet been exami­ned in detail. The A-to-I and C-to-U substitutions within the mitochondria of paradinids play a role in modifying RNA sequences to restore functional amino acids, which may be mediated by ADAR-like and PPR-DWY proteins. ADAR-like may have been inherited from LECA, and PPR-DWY protein may have been obtained by HGT. These results expand our knowledge of the diversity of RNA editing and contribute to our understanding of how RNA editing may have originated and evolved in both paradinids and eukaryotes.

### Data availability

The mitochondrial genomes of Paradinida spp. FC901 and SRM-001 are available under GenBank accession numbers LC733240 and LC733241, respectively. Raw sequencing data for genome reconstruction and the confirmation of RNA editing are available under GenBank BioProject accession number PRJDB14367. Their 18S rRNA gene sequences are available under GenBank accession numbers LC730879 (FC901) and LC730880 (SRM-001). The ADAR sequences that were newly detected and analyzed in the present study as well as the datasets may be found in the online repository Dryad at https://doi.org/10.5061/dryad.mcvdnck4z.

## Citation

Yabuki, A., Fujii, C., Yazaki, E., Tame, A., Mizuno, K., Obayashi, Y., and Takao, Y. (2025) Massive RNA Editing in Ascetosporean Mitochondria. *Microbes Environ ***40**: ME24070.

https://doi.org/10.1264/jsme2.ME24070

## Supplementary Material

Supplementary Material 1

Supplementary Material 2

Supplementary Material 3

## Figures and Tables

**Table 1.** Summary of synonymous and nonsynonymous substitutions in protein-coding genes and ORFs.

Strain	Gene	Length (bp)*^1^	Subs*^2^	Syn-Subs	Nonsyn-Subs	Ka	Ks	Ka/Ks
FC901	*atp1*	1,167	32	0	32	0.0361974	NA	NA
	*nad7*	1,209	38	0	38	0.0410346	NA	NA
	*nad2*	1,107	4	0	4	0.00458467	NA	NA
	*orf549*	1,647	3	0	3	0.0023016	NA	NA
	*orf174*	516	4	0	4	0.00952518	NA	NA
	*orf179*	537	0	0	NA	NA	NA	NA
	*orf101*	303	1	0	1	0.00414862	NA	NA
	*nad4*	1,374	26	0	26	0.0244923	NA	NA
	*nad1*	1,140	24	0	24	0.0274965	NA	NA
	*cox3*	936	20	2*^3^	18	0.0245131	0.0106216	2.30786
	*cox2*	396	6	0	6	0.0193405	NA	NA
	*cob*	909	20	0	20	0.0290719	NA	NA
	*atp9*	225	2	0	2	0.0117923	NA	NA
	*cox1*	1,533	54	0	54	0.0472416	NA	NA
	*orf471*	1,413	3	0	3	0.00268313	NA	NA
	*nad5*	1,377	19	0	19	0.0180025	NA	NA
	*atp6*	675	22	0	22	0.0435322	NA	NA
	*nad3*	417	7	0	7	0.0219111	NA	NA
SRM-001	*nad3*	420	7	0	7	0.0214919	NA	NA
	*cob*	912	18	0	18	0.0260917	NA	NA
	*cox1*	1,440	45	0	45	0.0415608	NA	NA
	*nad5*	1,362	19	0	19	0.0182314	NA	NA
	*orf174*	522	0	0	NA	NA	NA	NA
	*orf172*	513	2	0	2	0.00487225	NA	NA
	*nad6*	996	3	0	3	0.00382167	NA	NA
	*nad7*	1,209	35	0	35	0.0376654	NA	NA
	*orf103*	309	1	0	1	0.004035	NA	NA
	*atp9*	231	0	0	NA	NA	NA	NA
	*nad4*	1,353	27	0	27	0.0258708	NA	NA
	*atp6*	648	21	0	21	0.043372	NA	NA
	*atp1*	1,179	30	0	30	0.033471	NA	NA
	*cox3*	603	13	0	13	0.0280078	NA	NA
	*nad2*	1,074	3	0	3	0.0035415	NA	NA
	*cox2*	393	8	0	8	0.0262	NA	NA
	*nad1*	984	23	0	23	0.0308701	NA	NA

*^1^ Three bases comprising stop codons were eliminated from ‘length’ due to software settings. *^2^ Substitutions, the editing level of which was more than 50%, were counted and analyzed. Substitutions in stop codons were not included because they were eliminated in the preanalytical process ^(^*^1)^. *^3^ These two sites exited in the CDS overlapped by *cox3* and *cox2*, and were nonsynonymous substitutions in *cox2*.
