# Supplementary material for: Massive RNA Editing in Ascetosporean Mitochondria
Source: Microbes Environ. 2025 Mar 15;40(1):ME24070. doi: 10.1264/jsme2.ME24070 (PMC11946409; doi:10.1264/jsme2.ME24070)
Supplement: Supplementary file 1 — Supplementary Material 1 [file 40_24070_s1.docx]

**SUPPLEMENTARY INFORMATION**

**Supplementary materials and methods**

***Scanning electron microscopy***

Axenic cells grown on a glass slide were fixed with 2.5% glutaraldehyde in the cultivation medium at 4 °C. Fixed cells were washed using 0.22 µm filtered artificial seawater (FASW, 3.5% Rei-Sea Marine II; Iwaki Co. Ltd., Tokyo, Japan) and postfixed with 2% osmium tetroxide dissolved in FASW for 2 h. Postfixed cells were dehydrated using a graded ethanol series, dried using a JCPD-5 critical point drying device (JEOL, Akishima, Japan), and coated with osmium using an OPC-80 osmium coater (Filgen, Nagoya, Japan). The specimens were imaged using a field-emission scanning electron microscope (Quanta 450 FEG; Thermo Fisher Scientific) operating at 5 kV.

***Western blot analysis***

Approximately 200 mL mid-exponential-phase cell culture of Paradinida sp. FC901 was centrifuged at 2,400 × *g* for 5 min. The cell pellet was frozen and sent to Genostuff Co., Ltd. (Tokyo, Japan), where the following experiments were conducted.

The frozen cell pellet was homogenized in RIPA buffer (Fujifilm, Osaka, Japan) containing 1/100 (v/v) Protease Inhibitor Cocktail (Merck) for 30 min at 4 °C. After centrifugation at 18,000 × *g* for 5 min at 4 °C, the aqueous phase was recovered and utilized for initial protein assay using a BCA Protein Assay Kit (Thermo Fisher Scientific). Proteins (30 µg) were mixed with a sample buffer (Thermo Fisher Scientific) and a Sample Reducing Agent (Thermo Fisher Scientific) and separated by 5–20% gradient polyacrylamide gel electrophoresis. Separated proteins were transferred onto a polyvinylidene difluoride membrane (ATTO, Tokyo, Japan), and the membrane was blocked for 1 h at room temperature with 5% skim milk in Tris-buffered saline containing 0.1% Tween-20 (TBST). The membrane was then incubated overnight at 4 °C with a polyclonal anti-ADAR antibody HPA051519 (Merck) diluted to 1:500 in 0.1% TBST containing 5% bovine serum albumin. The membrane was washed four times with 0.1% TBST and incubated for 1 h at room temperature with horseradish peroxidase-conjugated anti-rabbit IgG (#7074; Cell Signaling Technology, Danvers, MA, USA) diluted 1:5,000 with 0.05% TBST containing 5% skimmed milk. After washing the membrane four times with 0.05% TBST, immunoreactive bands were visualized using Immobilon (Merck) and recorded on a C-DiGit (LI-COR, Lincoln, NE, USA).

**Supplementary discussion**

***Taxonomy of Paradinida spp. FC901 and SRM-001***

Order Paradinida (paradinids) currently includes only one genus, *Paradinium* Chatton, 1910 (Chatton*,* 1910), and three species (Chatton and Soyer*,* 1973). As all the described species are parasites of copepods, several unidentified species that are possibly new have been reported from different copepod species (Chatton*,* 1920; Skovgaard and Daugbjerg*,* 2008). Although their complete life cycles are not understood, they have been estimated based on the findings of partial cell stages. Amoeboid cells grow in the body cavity of host copepods, and dividing cells are interconnected by fine pseudopodia. After growth in the body cavity, cells move to the lumen of the host intestine and are excreted through the anus. Released cells form an outstanding cyst, called a gonosphere, at the urosome of the host. Characteristics of the gonosphere, such as color, size, and number of chromosomes, are mainly utilized in their taxonomy (Chatton*,* 1910; Chatton and Soyer*,* 1973). Flagellated cells hatch from the gonosphere and probably re-parasitize another host; however, this has not yet been confirmed and the possibility of a secondary host has not been ruled out.

Cultures of FC901 and SRM-001 were established from water samples, not from copepods. Both strains grew axenically in Hemi medium and showed network-forming amoeboid cells (Figs. S1, S2). No other stages, such as flagellates or cysts, were observed. Amoeboid cells were similar to those of paradinids, and they phylogenetically branched within the Paradinida clade with high statistical support. However, as gonospheres were not observed, and their characteristics could not be compared with those of the described species, their taxonomic identification remains unclear. FC901 and SRM-001 were phylogenetically distinct in the clade of Paradinida, and their morphological characteristics were slightly different; almost all FC901 cells had microvilli-like structures on the surface, whereas they were rarely observed in SRM-001 cells. This difference may be useful for distinguishing between them, although cells with and without microvilli-like structures were also found in the cultures of FC901 and SRM-001 (Figs. S1G, S2F). Therefore, we conclude that their proper identification and taxonomic discussion to distinguish them should be revisited with further data, and at present, they should be described only as Paradinida sp. FC901 and SRM-001.

**References**

Chatton, E. (1910) Protozoaires parasites des branchies des labres. *Amoeba Mucicola* 239–266.

Chatton, E. (1920) Les Péridiniens parasites: morphologie, reproduction, ethologie. *Arch Zool Exp Gén* **59**, 1–475

Chatton, E., and Soyer, M. (1973) Le cycle évolutif de *Paradinium poucheti* Chatton, flagellé parasite plasmodial des copépodes les paradinides. *Ann Sci Nat Zool* **12**: 27–60.

Flati, T., Gioiosa, S., Spallanzani, N., Tagliaferri, I., Diroma, M.A., Pesole, G., *et al*. (2020) HPC-REDItools: a novel HPC-aware tool for improved large scale RNA-editing analysis. *BMC Bioinformatics* **21(Suppl 10)**: 353.

Kim, D., Paggi, J.M., Park, C., Bennett, C., and Salzberg, S.L. (2019) Graph-based genome alignment and genotyping with HISAT2 and HISAT-genotype. *Nat Biotechnol* **37**: 907–915.

Skovgaard, A., and Daugbjerg, N. (2008) Identity and systematic position of *Paradinium poucheti* and other Paradinium-like parasites of marine copepods based on morphology and nuclear-encoded SSU rDNA. *Protist* **159**: 401–413.

Zhang, Z. (2022) KaKs_Calculator 3.0: calculating selective pressure on coding and non-coding sequences. *Genomics Proteomics Bioinformatics* **20**: 536–540.

**Supplementary figures**


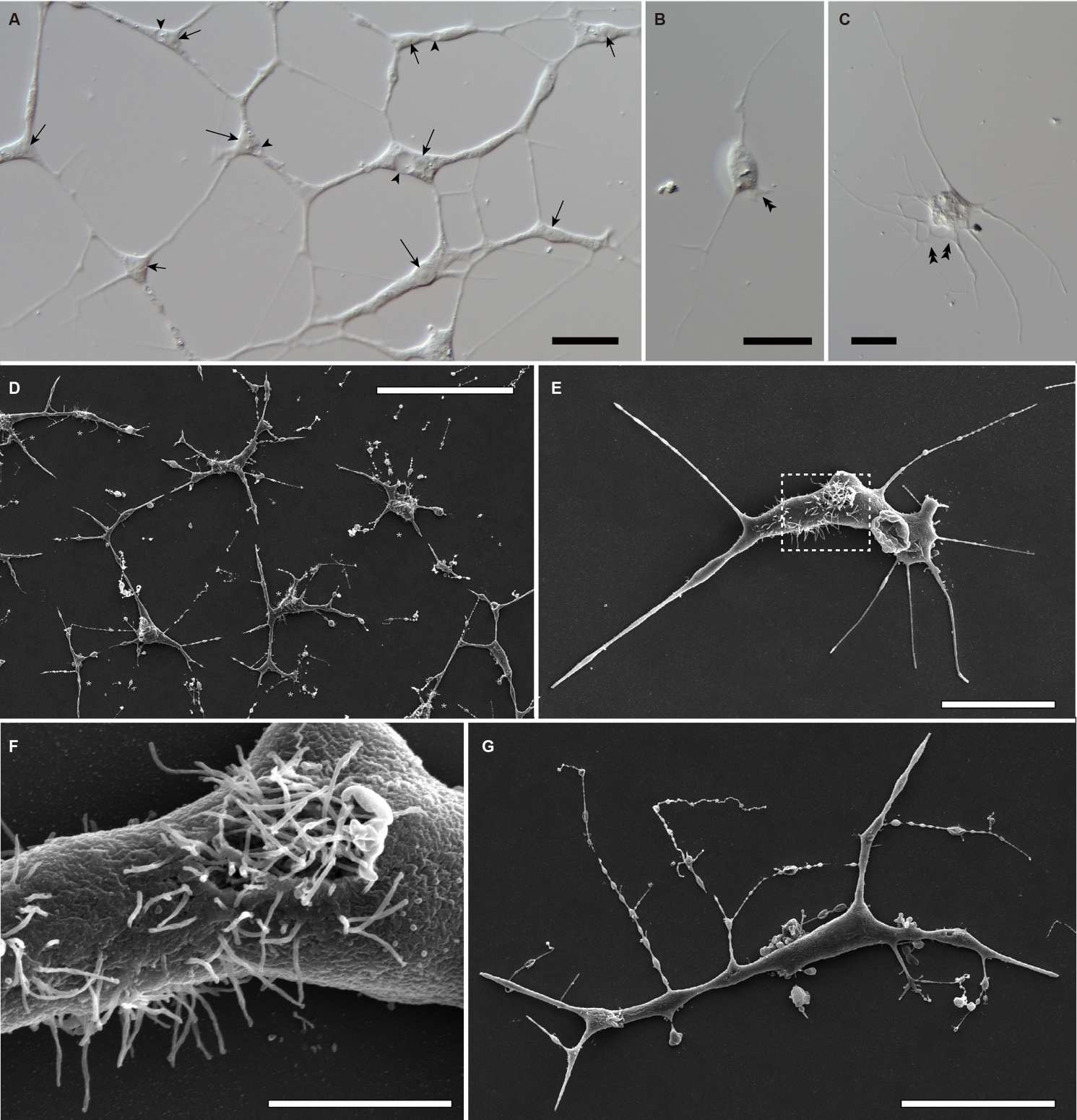


**Fig. S1.** Images of Paradinida sp. FC901 cells in the mid-exponential phase. **A.** Differential interference contrast (DIC) image of the network-forming cells. Each cell containing a single nucleus (arrow) is connected to the neighboring cells with thin filopodia. A clear vacuole (arrowhead) occasionally exists in cells. Scale bar, 20 µm. **B and C.** DIC images of the cell with a lobate pseudopodium (double arrowhead). Scale bar, 20 µm. **D.** Scanning electron microscopic (SEM) image of network-forming cells. The connecting filopodia were broken by the fixation artifact. Cells marked by asterisks possess microvilli-like structures on the surface. Scale bar, 50 µm. **E.** High-magnification SEM image of the cell with the microvilli-like structure. Scale bar, 10 µm. **F.** Enlarged view of the microvilli-like structure enclosed by a white square in Fig. S1D. Scale bar, 3 µm. **G.** High-magnification SEM image of the cell without microvilli-like structure. Scale bar, 20 µm.


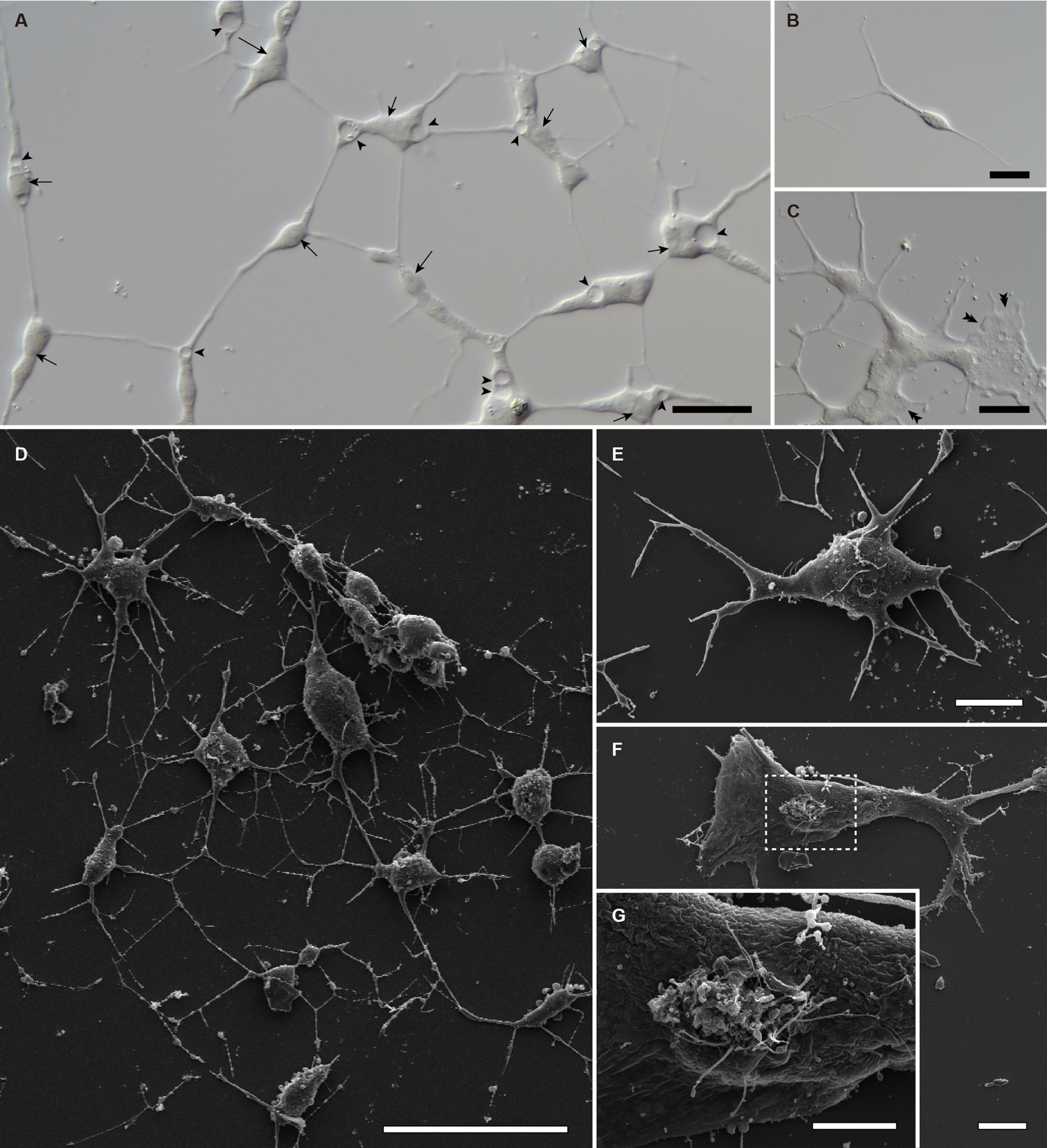


**Fig. S2.** Images of Paradinida sp. SRM-001 cells in the mid-exponential phase. **A.** DIC image of network-forming cells. Each cell containing a single nucleus (arrow) is connected with neighboring cells through thin filopodia. A clear vacuole (arrowhead) occasionally exists in cells. Scale bar, 20 µm. **B and C.** DIC images of the cell with a lobate pseudopodium (double arrowhead). Scale bar, 20 µm. **D.** SEM image of network-forming cells. Cells possessing microvilli-like structures on the surface are rarely recognized. Scale bar, 50 µm. **E.** High-magnification SEM image of the cell. Scale bar, 10 µm. **F.** High-magnification SEM image of the cell with microvilli-like structure. Scale bar, 20 µm. **G.** Enlarged view of the microvilli-like structure enclosed by a white square in Fig. S2F. Scale bar, 3 µm.


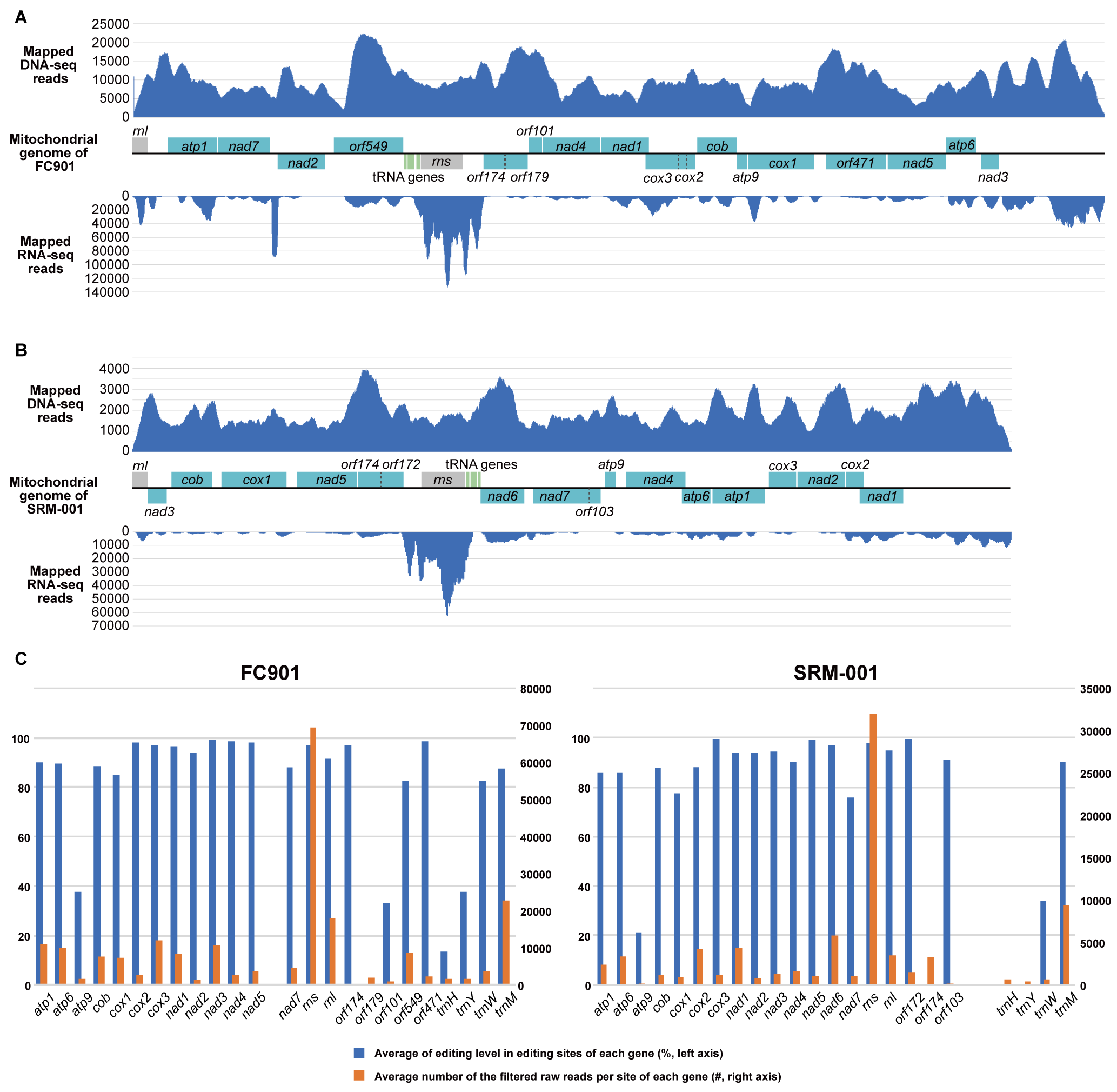


**Fig. S3.** Additional information about the mitochondrial genome reconstruction, and editing and transcriptional level of mitochondrial genes. **A, B.** Mapping results of the filtered reads of DNA-seq and RNA-seq data on the mitochondrial genome of FC901(A) and SRM-001 (B). **C.** Bar graphs summarizing the editing level and mapped read number of each gene.


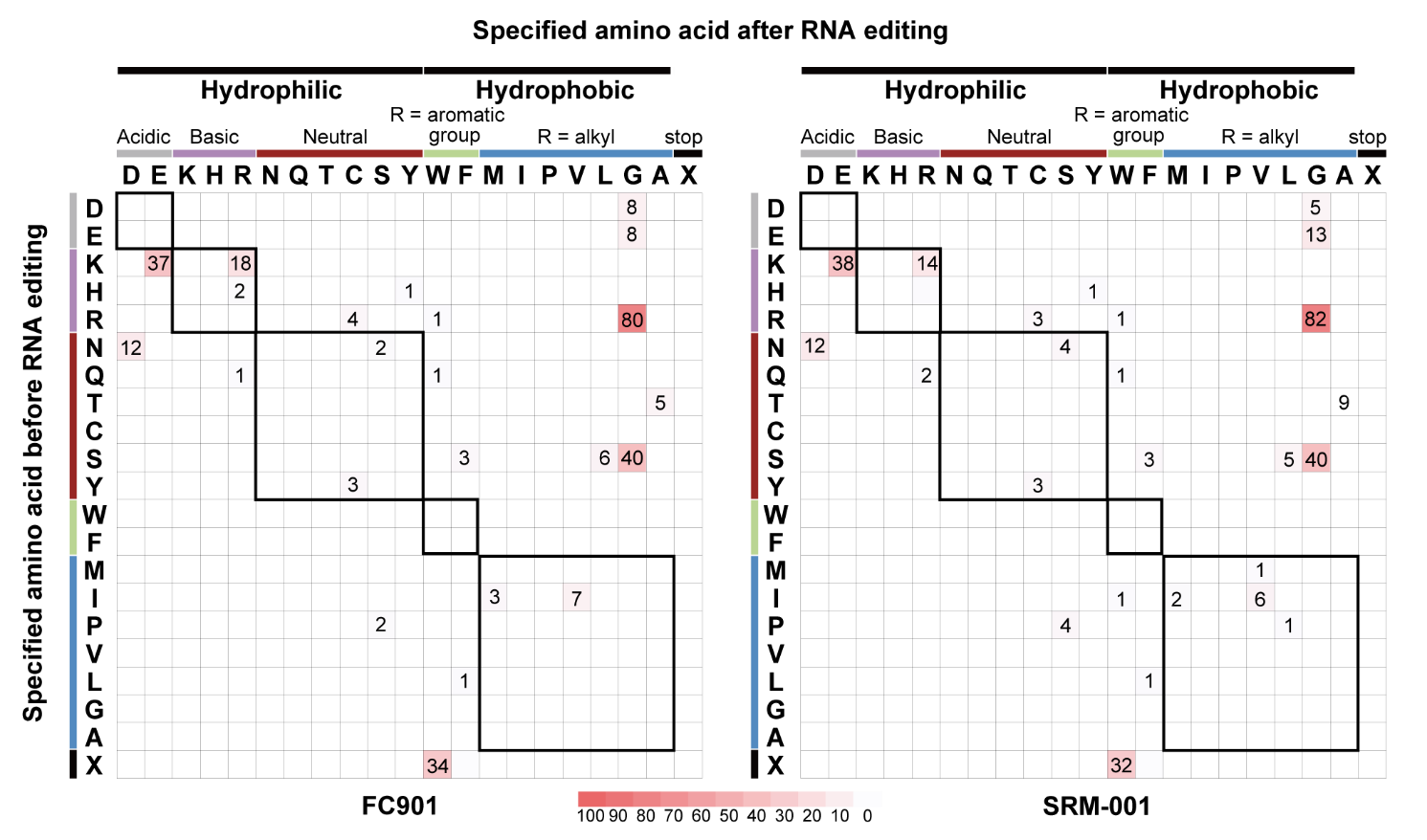


**Fig. S4.** Specified amino acids before and after RNA editing (editing level > 50%) in two paradinids. The number in each square indicates the number of the detected substitutions, which are also shown with heatmap color. Amino acids are represented by single letters. Substitutions in the same chemical groups are shown in bold-line squares.

**
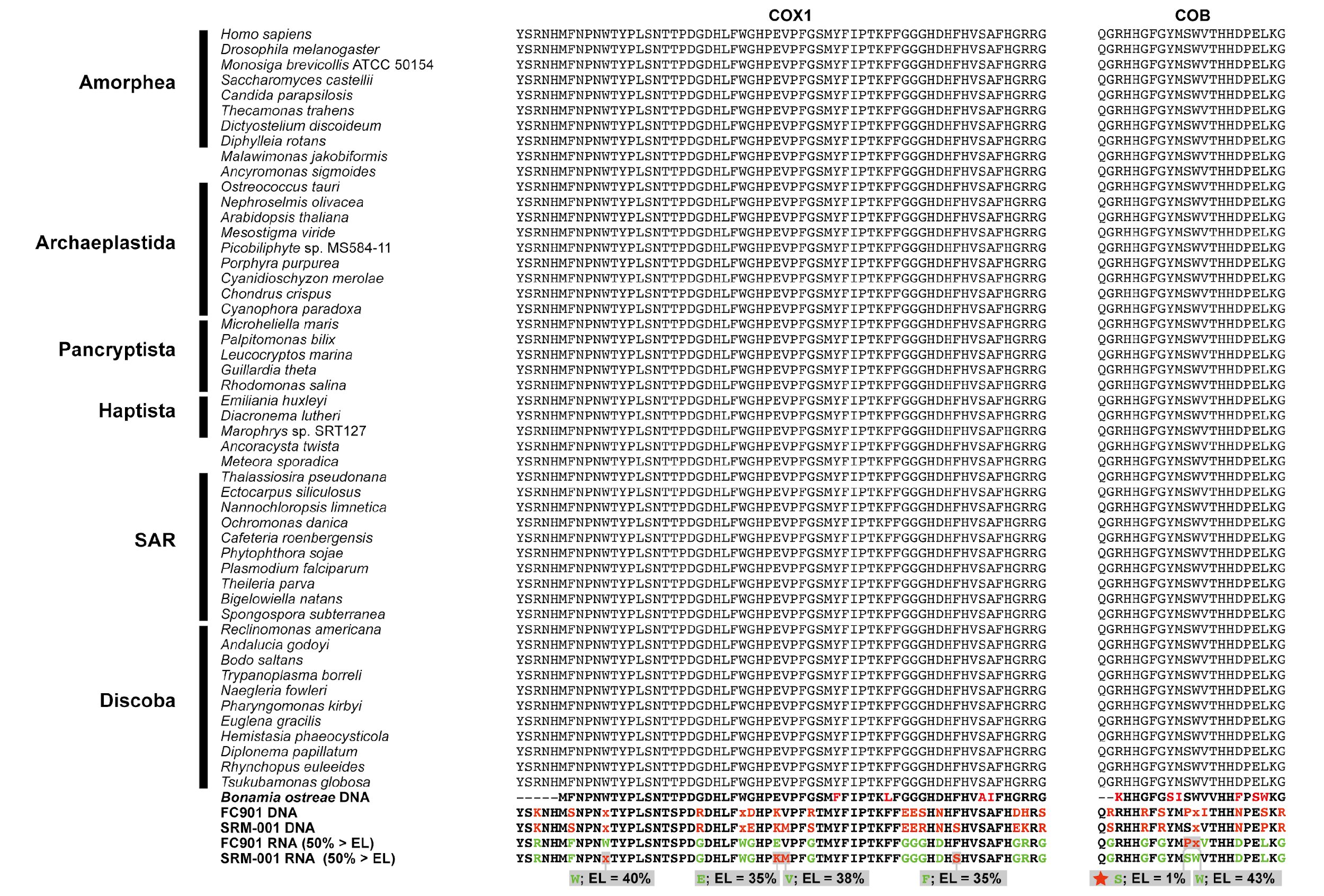
**

**Fig. S5.** The alignment of well-conserved amino acid (AA) residues in COX1 and COB sequences. The 62 and 22 AA residues shown here were completely conserved in whole eukaryotes except for ascetosporeans in our alignments of COX1 and COB, respectively. The mismatched AAs and the stop codons (x) detected in ascetosporean DNA and RNA sequences are shown in red. The AAs shown in green indicated that they were restored to the conserved AAs by RNA editing. Two positions in FC901 COB and four positions in SRM-001 COX1, respectively, were not restored when 50% of editing level (EL) was applied as the threshold. However, five of them were also restored at approximately 40% of editing level. Their editing levels were specifically shown in gray squares. At only one position in FC901 COB (marked with a red star), the editing level corresponding to the AA restoration was 1%. The AA residues that were estimated from the candidate of the partial mitochondrial genome of *Bonamioa ostreae* (Haplosporida) with translation codon table 4 (i.e., table for mold, protozoan, and coelenterate mitochondrial + *Mycoplasma* / *Spiroplasma*) added.


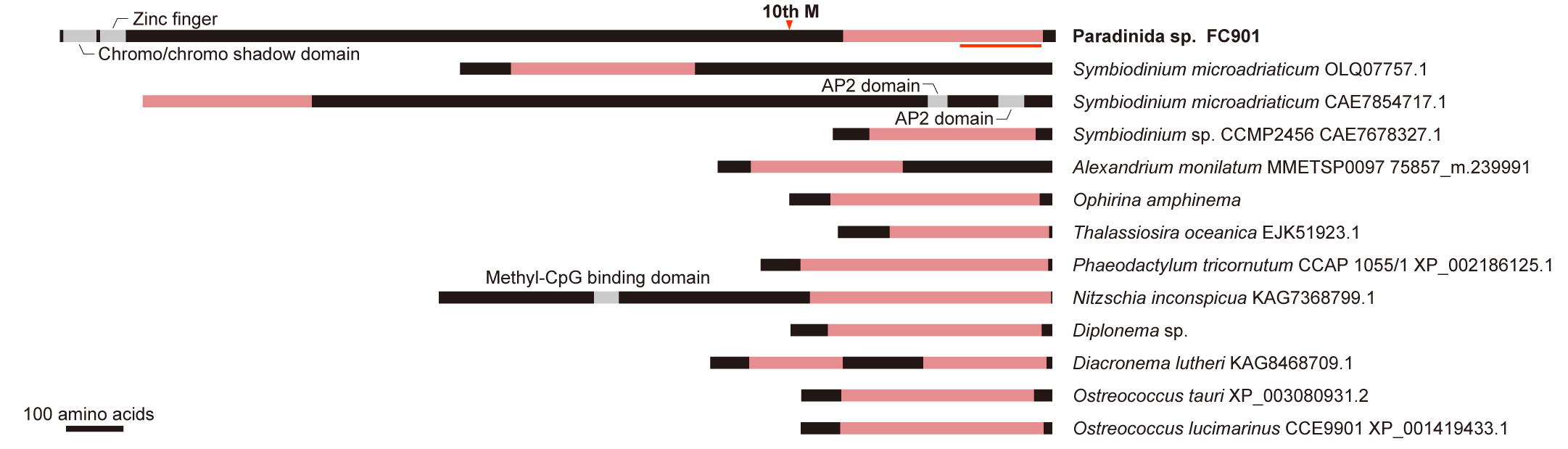


**Fig. S6.** Domain structures of the selected ADAR-like sequences. Pink bars indicate deaminase domains, with other detected domains indicated by dark grey bars. ADAR-like sequence of SRM-001 was very partial and is not shown here. The position of 10^th^ methionine is shown with a red arrowhead and antigenic region of anti-ADAR antibody HPA051519 are shown with a red bar.


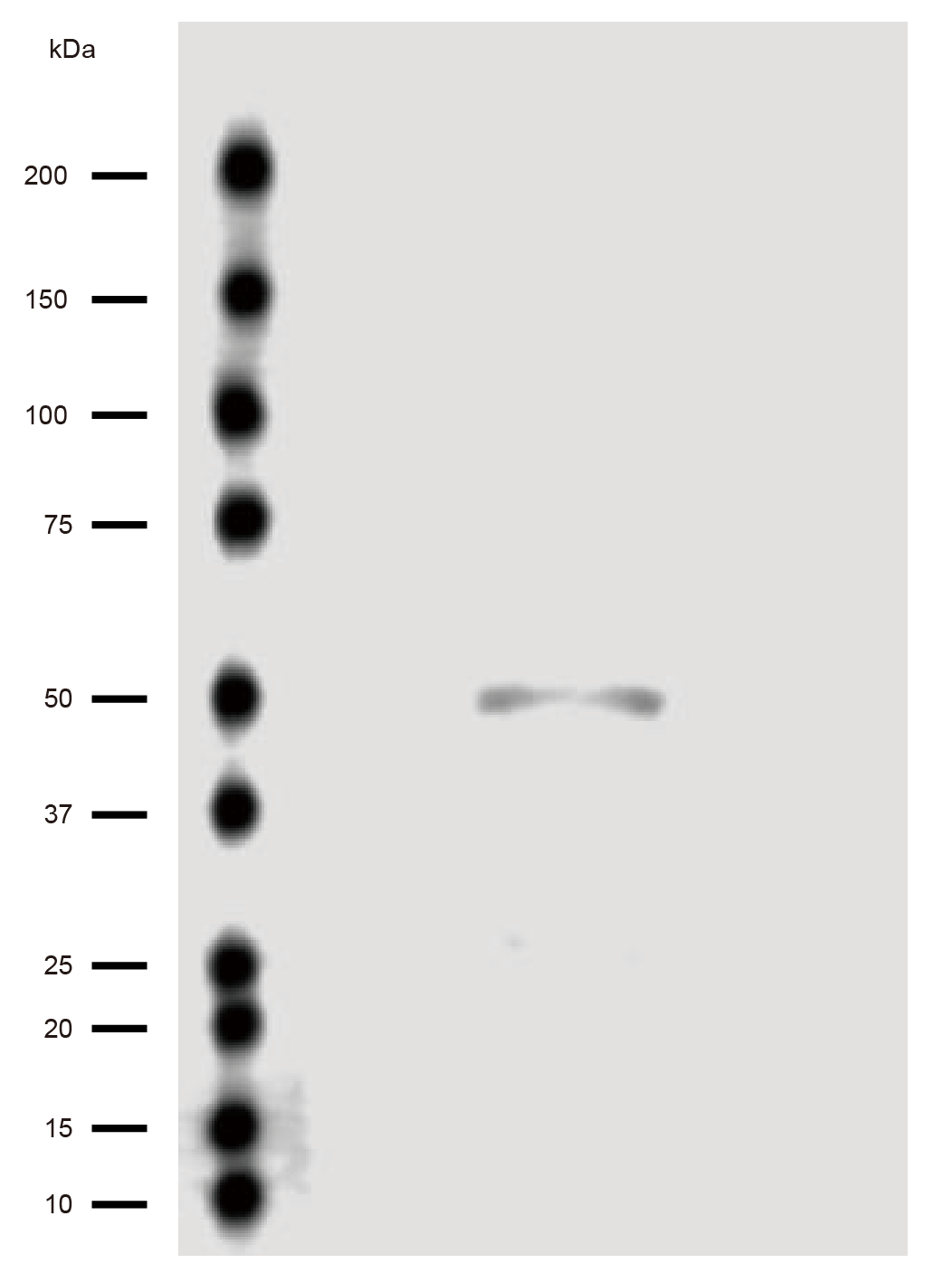


**Fig. S7.** Western blot analysis of anti-ADAR antibody HPA051519 with Paradinida sp. FC901, with a single band at approximately 50 kDa.

**Supplementary tables**

**Table S1.** Summary of the initial samples for establishing the cultures. The location, longitude, latitude, depth (m) and date are given for both cultures.

|  | Sampling area | Longitude (N) | Latitude (E) | Depth (m) | Date |
| --- | --- | --- | --- | --- | --- |
| Paradinida sp. FC901 | Tokyo Bay, Japan | 35.3194° | 139.6507° | Surface | November 16, 2019 |
| Paradinida sp. SRM-001 | Suruga Bay, Japan | 34.8833° | 138.6417° | 200 | January 15, 2019 |

**Table S2.** Summary of the sequencing analyses. The DRR number, the DNA and RNA extraction kits, the library kit, sequencing target, platform, reads, bases, Q30 (%), number of contigs, and BUSCO (%) are given for each.

| Organism | DRR no. | DNA/RNA  Extraction kit | Library kit | Sequencing target | Platform | Reads | Bases (Gb) | Q30 (%) | No. of contigs | BUSCO (%) |
| --- | --- | --- | --- | --- | --- | --- | --- | --- | --- | --- |
| Paradinida sp.  SRM-001 | DRR408102 | Invitrogen PureLink Genomic DNA Mini Kit | VAHTS Universal Pro DNA Library Prep Kit for Illumina | Genomic DNA | NovaSeq 6000 | 51,617,946 | 15.485 | 88.98 | - | - |
|  | DRR408103 | Qiagen RNeasy Mini Kit | NEBNext Ultra II Directional RNA Library Prep Kit for Illumina | mRNA | NovaSeq 6000 | 13,073,696 | 3.922 | 93.95 | 86,986 | 62.7 |
| Paradinida sp.  FC901 | DRR408104 | Invitrogen PureLink Genomic DNA Mini Kit | VAHTS Universal Pro DNA Library Prep Kit for Illumina | Genomic DNA | NovaSeq 6000 | 39,678,757 | 11.904 | 92.64 | - | - |
|  | DRR408105 | TRI Reagent, followed by RNA Clean & Concentrator Kits | NEBNext Ultra II Directional RNA Library Prep Kit for Illumina | mRNA | NovaSeq 6000 | 13,595,734 | 4.079 | 94.52 | 178,021 | 85.1 |
|  | DRR408106 | Qiagen RNeasy Mini Kit | MGIEasy RNA Directional Library Prep Set V2.0 | mRNA | DNBSEQ-G400 | 78,445,090 | 11.766 | 85.44 |  |  |
|  | DRR408107 | TRI Reagent, followed by RNA Clean & Concentrator Kits | NEBNext Ultra II Directional RNA Library Prep Kit for Illumina | mRNA | NovaSeq 6000 | 193,727,174 | 58.118 | 94.16 |  |  |

**Table S3.** Summary of the site number of each DNA heterogeneity in the mitochondrial genome of two paradinids. The rate of heterogeneity (%) and the number of sites are given for both samples.

| Strain | Rate of heterogeneity (%) | Number of sites |
| --- | --- | --- |
| FC901 | 26.6 | 1 |
|  | 7.3 | 1 |
|  | 2≥, >1 | 4 |
|  | 1≥, >0 | 4 |
|  | 0 | 23,038 (99.96%) |
|  | Total | 23,048 |
| SRM-001 | 7.0 | 1 |
|  | 4≥, >3 | 1 |
|  | 3≥, >2 | 2 |
|  | 2≥, >1 | 29 |
|  | 1≥, >0 | 175 |
|  | 0 | 19,891 (98.97%) |
|  | Total | 20,099 |

**Table S4**. Summary of mitochondrial RNA editing in Paradinida spp. FC901 and SRM-001.

Shown in separate excel file.

The number of the editing sites was counted applying the threshold to more than 50%, 30% or 10%. The highest and second highest editing density., i.e., the number of editing sites per gene length, are shown on orange and pale orange backgrounds in FC901 and green and pale green backgrounds, respectively.

* The editing density is calculated using the following formula:

$$Editing density= \frac{number of editing sites in a given gene \times100}{length of a given gene}$$

**Table S5.** Number of the editing sites at each codon position per level.

| Editing  level (%) | FC901 | | | SRM-001 | | |
| --- | --- | --- | --- | --- | --- | --- |
|  | codon1 | codon2 | codon3 | codon1 | codon2 | codon3 |
| 100≥, >90 | 190 | 71 | 4 | 165 | 56 | 2 |
| 90≥, >80 | 8 | 13 | 1 | 20 | 13 | 0 |
| 80≥, >70 | 7 | 5 | 0 | 7 | 7 | 0 |
| 70≥, >60 | 5 | 4 | 0 | 4 | 3 | 0 |
| 60≥, >50 | 5 | 1 | 0 | 6 | 5 | 0 |
| 50≥, >40 | 3 | 4 | 0 | 5 | 0 | 0 |
| 40≥, >30 | 8 | 3 | 0 | 5 | 10 | 0 |
| 30≥, >20 | 5 | 1 | 0 | 6 | 6 | 1 |
| 20≥, >10 | 1 | 3 | 0 | 5 | 2 | 0 |
| 10≥, >0 | 33 | 40 | 50 | 34 | 31 | 53 |
| 0 | 5427 | 5547 | 5637 | 4510 | 4634 | 4711 |

**Table S6.** Summary of editing level and mapped read number of RNA-seq of each gene. The editing level average (%) and mapped read number average are given for each gene for FC901 and SRM-001.

| Gene | FC901 | | SRM-001 | |
| --- | --- | --- | --- | --- |
|  | Editing level  average (%)* | Mapped read number average (#)** | Editing level  average (%)* | mapped read number average (#)** |
| *atp1* | 89.8 | 11067.3 | 85.8 | 2397.9 |
| *atp6* | 89.5 | 10008.0 | 85.9 | 3288.6 |
| *atp9* | 37.7 | 1470.9 | 21.0 | 84.0 |
| *cob* | 88.3 | 7540.8 | 87.5 | 1075.7 |
| *cox1* | 84.7 | 7380.6 | 77.6 | 880.5 |
| *cox2* | 98.1 | 2633.7 | 87.9 | 4230.0 |
| *cox3* | 96.9 | 12145.6 | 99.3 | 1130.7 |
| *nad1* | 96.3 | 8326.4 | 93.8 | 4287.6 |
| *nad2* | 94.0 | 1136.4 | 93.8 | 684.4 |
| *nad3* | 99.1 | 10724.5 | 94.6 | 1274.3 |
| *nad4* | 98.3 | 2708.9 | 90.3 | 1625.3 |
| *nad5* | 98.0 | 3667.0 | 98.9 | 958.2 |
| *nad6* (SRM-001 only) |  |  | 97.0 | 5822.7 |
| *nad7* | 88.1 | 4534.6 | 75.9 | 964.2 |
| *rns* | 97.0 | 69410.4 | 97.9 | 31980.4 |
| *rnl* | 91.3 | 18058.2 | 94.6 | 3406.6 |
| *orf174* (*orf172* of SRM-001) | 97.0 | 377.9 | 99.7 | 1449.4 |
| *orf179* (*orf174* of SRM-001) | NA | 2102.6 | NA | 3268.3 |
| *orf101* (*orf103* of SRM-001) | 33.0 | 1066.1 | 91.0 | 164.1 |
| *orf549* (FC901 only) | 82.3 | 8620.7 |  |  |
| *orf471* (FC901 only) | 98.3 | 2238.2 |  |  |
| *trnH* | 13.5 | 1519.0 | NA | 620.4 |
| *trnY* | 37.5 | 1497.2 | NA | 406.0 |
| *trnW* | 82.6 | 3586.0 | 33.8 | 622.3 |
| *trnM* | 87.5 | 22887.0 | 90.0 | 9393.9 |
| *r* | 0.261613172 | | 0.237952855 | |
| *t* | 1.271352174 | | 1.122684155 | |
| *p* | 0.216880991 | | 0.274243922 | |

‘*r’, ‘t’, and ‘p*’ at the lowest three rows indicate the results of statistical analysis for correlation between editing level average and mapped read number average (= transcriptional level).

*The editing level average of each gene was calculated from the sites that were detected at more than 10% editing level threshold. Sites that were not involved in RNA editing were excluded from the analysis.

** The mapped read average is calculated using the following formula:

Mapping read average = number of total raw reads mapping on a given gene / length of a given gene.

**Table S7.** Results of Mitofates analyses.

| Gene | Organism | contig ID or accession number | Probability of mitochondrial presequence |
| --- | --- | --- | --- |
| PPR-DYW | Paradinida sp. FC901 | TRINITY_DN317_c0_g3_i1 | 0.717 |
|  | *Crepidotus variabilis* | KAF9528688.1 | 0.997 |
|  | *Galerina marginata* CBS 339.88 | KDR72683.1 | 0.984 |
|  | *Flammula alnicola* | KAF8954993.1 | 0.983 |
|  | *Hebeloma cylindrosporum* h7 | KIM46640.1 | 0.934 |
|  | *Laccaria amethystina* LaAM-08-1 | KIK00860.1 | 0.87 |
|  | *Amanita rubescens* | KAF8337132.1 | 0.669 |
|  | *Piloderma croceum* F 1598 | KIM77073.1 | 0.68 |
|  | *Laccaria bicolor* S238N-H82 | XP_001886834.1 | 0.031 |
|  | *Amanita phalloides* | KAK2461236.1 | 0.285 |
| PPR protein | Paradinida sp. FC901 | TRINITY_DN11807_c1_g1_i1 | 1 |
|  | Paradinida sp. FC901 | TRINITY_DN11442_c0_g1_i1 | 0.997 |
|  | Paradinida sp. FC901 | TRINITY_DN7867_c1_g1_i6 | 0.994 |
|  | Paradinida sp. FC901 | TRINITY_DN4182_c0_g1_i13 | 0.99 |
|  | Paradinida sp. FC901 | TRINITY_DN4182_c0_g1_i15 | 0.99 |
|  | Paradinida sp. FC901 | TRINITY_DN4182_c0_g1_i22 | 0.99 |
|  | Paradinida sp. FC901 | TRINITY_DN4182_c0_g1_i4 | 0.99 |
|  | Paradinida sp. FC901 | TRINITY_DN4182_c0_g1_i5 | 0.99 |
|  | Paradinida sp. FC901 | TRINITY_DN4182_c0_g1_i9 | 0.99 |
|  | Paradinida sp. FC901 | TRINITY_DN2700_c0_g2_i1 | 0.987 |
|  | Paradinida sp. FC901 | TRINITY_DN62929_c0_g1_i1 | 0.961 |
|  | Paradinida sp. FC901 | TRINITY_DN7867_c1_g1_i2 | 0.953 |
|  | Paradinida sp. FC901 | TRINITY_DN4182_c0_g1_i20 * | 0.566 |
|  | Paradinida sp. FC901 | TRINITY_DN4182_c0_g1_i17 * | 0.153 |
|  | Paradinida sp. FC901 | TRINITY_DN7352_c0_g2_i1 | 0.058 |
|  | Paradinida sp. FC901 | TRINITY_DN7352_c0_g1_i2 | 0.023 |
|  | Paradinida sp. FC901 | TRINITY_DN2579_c4_g3_i1 | 0.013 |
|  | Paradinida sp. FC901 | TRINITY_DN2579_c4_g1_i10 | 0.008 |
|  | Paradinida sp. FC901 | TRINITY_DN4182_c0_g1_i19 | 0.008 |
|  | Paradinida sp. FC901 | TRINITY_DN4182_c0_g1_i6 | 0.008 |
|  | Paradinida sp. FC901 | TRINITY_DN2579_c4_g1_i16 | 0.006 |
|  | Paradinida sp. FC901 | TRINITY_DN7352_c0_g1_i5 | 0.006 |
|  | Paradinida sp. FC901 | TRINITY_DN7352_c0_g1_i7 | 0.006 |
|  | Paradinida sp. FC901 | TRINITY_DN7352_c0_g1_i1 | 0.004 |
|  | Paradinida sp. FC901 | TRINITY_DN10504_c0_g1_i1 | 0.003 |
|  | Paradinida sp. FC901 | TRINITY_DN20122_c0_g1_i1 | 0.002 |
|  | Paradinida sp. FC901 | TRINITY_DN20122_c0_g1_i2 | 0.002 |
|  | Paradinida sp. FC901 | TRINITY_DN20122_c0_g1_i3 | 0.002 |
|  | Paradinida sp. FC901 | TRINITY_DN2579_c4_g1_i11 | 0.002 |
|  | Paradinida sp. FC901 | TRINITY_DN2579_c4_g1_i12 | 0.002 |
|  | Paradinida sp. FC901 | TRINITY_DN2579_c4_g1_i14 | 0.002 |
|  | Paradinida sp. FC901 | TRINITY_DN2579_c4_g1_i7 | 0.002 |
|  | Paradinida sp. FC901 | TRINITY_DN2579_c4_g1_i9 | 0.002 |
|  | Paradinida sp. FC901 | TRINITY_DN4182_c0_g1_i10 | 0.002 |
|  | Paradinida sp. FC901 | TRINITY_DN4182_c0_g1_i16 | 0.002 |
|  | Paradinida sp. FC901 | TRINITY_DN6977_c1_g2_i1 * | 0.002 |
|  | Paradinida sp. FC901 | TRINITY_DN7352_c0_g3_i2 * | 0.002 |
|  | Paradinida sp. FC901 | TRINITY_DN12304_c0_g1_i1 | 0.001 |
|  | Paradinida sp. FC901 | TRINITY_DN4182_c0_g1_i18 | 0.001 |
|  | Paradinida sp. FC901 | TRINITY_DN6977_c0_g1_i1 | 0.001 |
|  | Paradinida sp. FC901 | TRINITY_DN6977_c1_g1_i1 * | 0.001 |
|  | Paradinida sp. FC901 | TRINITY_DN289_c3_g1_i3 | 0 |
| ADAR-like | Paradinida sp. FC901 | TRINITY_DN2269_c0_g1_i3 | 0.001 |
|  | Paradinida sp. FC901 | TRINITY_DN2269_c0_g1_i3 (from 10th M) | 0.104 |

In the sequences indicated with a single asterisk, a methionine was not detected in the first 50 amino acids and they may have been sequenced partially. Those sequences were directly subjected to MitoFates analysis without trimming the N´ region before first methionine.

PPR-DYW protein of *Hygrocybe coccinea* was found from its genome sequence and full length of its PPR-DYW protein could not be determined certainly. Its sequence was omitted from MitoFates analysis.

The scores with green and gray backgrounds are corresponding to positive and negative results of the existence of mitochondrial presequence, respectively.

**Table S8.** List of ADAR, ADAR-like, and ADAT sequences analyzed in this study.

|  |  |  |  |  | **ADAR in the initial dataset tree** | **ADAT in the initial dataset tree** | **pADAR in the main dataset tree** | **ADAT in the main dataset tree** |
| --- | --- | --- | --- | --- | --- | --- | --- | --- |
| Diaphretickes | CAM clade | Archaeplastida | Viridiplantae | *Chlamydomonas reinhardtii* |  |  |  |  |
|  |  |  |  | *Chlorokybus atmophyticus* |  | 1 |  | 1 |
|  |  |  |  | *Mesostigma viride* |  |  |  |  |
|  |  |  |  | *Physcomitrium patens* |  | 1 |  | 1 |
|  |  |  |  | *Ulva flexuosa* |  |  |  |  |
|  |  |  |  | *Ostreococcus lucimarinus* | 1 |  | 1 |  |
|  |  |  |  | *Ostreococcus tauri* | 1 | 1 | 1 |  |
|  |  |  |  | *Marchantia polymorpha* |  | 1 |  | 1 |
|  |  |  |  | *Physcomitrium patens* |  | 1 |  | 1 |
|  |  |  |  | *Papaver bracteatum* |  | 1 |  | 1 |
|  |  |  |  | *Cypripedium flavum* |  | 1 |  | 1 |
|  |  |  |  | *Coffea eugenioides* |  | 1 |  | 1 |
|  |  |  |  | *Arabidopsis suecica* |  | 1 |  | 1 |
|  |  |  |  | *Arabidopsis thaliana* |  | 1 |  | 1 |
|  |  |  |  | *Prunus persica* |  | 1 |  | 1 |
|  |  |  |  | *Ziziphus jujuba* |  | 1 |  | 1 |
|  |  |  |  | *Syzygium oleosum* |  | 1 |  | 1 |
|  |  |  |  | *Rhododendron griersonianum* |  | 1 |  | 1 |
|  |  |  | Rhodophyta | *Rhodelphis limneticus* | 1 |  |  | 1* |
|  |  |  |  | *Rhodelphis marinus* | 1 |  |  | 1* |
|  |  |  |  | *Cyanidioschyzon merolae* |  |  |  |  |
|  |  |  |  | *Galdieria sulphuraria* |  |  |  |  |
|  |  |  |  | *Chondrus crispus* |  |  |  |  |
|  |  |  | Glaucophyta | *Cyanophora paradoxa* |  |  |  |  |
|  |  | Pancryptista |  | *Microheliella maris* |  |  |  |  |
|  |  |  |  | *Guillardia theta* | 1 | 1 |  |  |
|  |  |  |  | *Hemiarma marina* | 1 |  |  |  |
|  |  |  |  | *Goniomonas avonlea* |  | 1 |  |  |
|  | Telonema |  |  | *Telonema* sp. P-2 | 2 |  |  |  |
|  | SAR | Alveolata | Cilliate | *Oxytricha trifallax* |  |  |  |  |
|  |  |  |  | *Paramecium caudatum* |  |  |  |  |
|  |  |  | Dinoflagellate | *Pyrodinium bahamense* | 1 | 1 |  | 1 |
|  |  |  |  | *Scrippsiella trochoidea* CCMP3099 | 2 | 2 |  |  |
|  |  |  |  | *Alexandrium monilatum CCMP3105* | 2 | 1 | 1 | 1 |
|  |  |  |  | *Symbiodinium microadriaticum* | 2 |  | 2 |  |
|  |  |  |  | *Symbiodinium* sp. CCMP2456 | 1 | 1 | 1 | 1 |
|  |  |  |  | *Symbiodinium* sp. CCMP2592 |  | 1 |  | 1 |
|  |  |  |  | *Symbiodinium pilosum* |  | 1 |  | 1 |
|  |  |  | Apicomplexa | *Plasmodium falciparum* |  |  |  |  |
|  |  | Stramenopile |  | *Phaeodactylum tricornutum* | 1 | 1 | 1 | 1 |
|  |  |  |  | *Thalassiosira oceanica* | 1 | 1 | 1 | 1 |
|  |  |  |  | *Nitzschia inconspicua* | 1 | 1 | 1 | 1 |
|  |  |  |  | *Fistulifera solaris* |  | 1 |  | 1 |
|  |  |  |  | *Blastocystis sp.* |  | 1 |  | 1 |
|  |  |  |  | *Blastocystis hominis* |  | 1 |  | 1 |
|  |  |  |  | *Phytophthora megakarya* |  | 1 |  | 1 |
|  |  |  |  | *Albugo candida* |  | 1 |  | 1 |
|  |  | Rhizaria |  | *Bigelowiella natans* | 1 |  |  |  |
|  |  |  |  | *Plasmodiophora brassicae* |  |  |  |  |
|  |  |  |  | *Paulinella sp.* | 1 |  |  |  |
|  |  |  |  | *Paradinium* sp. FC901 | 1 | 1 | 1 |  |
|  |  |  |  | *Paradinium* sp. SRM | 1 | 1 | 1 |  |
|  |  |  |  | *Eucyrtidium acuminatum* | 1 |  |  |  |
|  | Haptophyta |  |  | *Chrysochromulina rotalis* | 1 |  |  |  |
|  |  |  |  | *Emiliania huxleyi* |  | 2 |  |  |
|  |  |  |  | *Diacronema lutheri* | 1 |  | 1 |  |
|  | Centrohelida |  |  | *Marophrys* sp. SRT127 |  | 1 |  | 1 |
|  |  |  |  | *Choanocystis* sp. FB-2015 | 1 |  |  |  |
|  |  |  |  | *Raineriophrys erinaceoides* |  |  |  |  |
|  | *Ancoracysta* |  |  | *Ancoracysta twista* |  |  |  |  |
|  | Hemimastigophra |  |  | *Hemimastix kukwesjijk* |  |  |  |  |
|  |  |  |  | *Spironema cf. multiciliatum* |  |  |  |  |
| Amorphea | Amoebozoa |  |  | *Acanthamoeba castellanii* |  | 1 |  | 1 |
|  |  |  |  | *Dictyostelium discoideum* |  | 1 |  | 1 |
|  |  |  |  | *Entamoeba histolytica* |  | 1 |  |  |
|  | Opisthokonts | Holozoa |  | *Capsaspora owczarzaki* |  | 1 |  | 1 |
|  |  |  |  | *Abeoforma whisleri* |  |  |  |  |
|  |  |  |  | *Creolimax fragrantissima* | 2 |  |  | 1** |
|  |  |  | Metazoa | *Homo sapiens* | 3 | 1 | 3 | 1 |
|  |  |  |  | *Mus musculus* | 3 | 1 | 3 | 1 |
|  |  |  |  | *Xenopus tropicalis* | 3 | 1 | 3 | 1 |
|  |  |  |  | *Gallus gallus* | 3 | 1 | 3 | 1 |
|  |  |  |  | *Danio rerio* | 3 | 1 | 3 | 1 |
|  |  |  |  | *Ciona intestinalis* | 2 | 1 | 2 | 1 |
|  |  |  |  | *Strongylocentrotus purpuratus* | 2 | 1 | 2 | 1 |
|  |  |  |  | *Amphimedon queenslandica* | 2 | 1 | 2 | 1 |
|  |  | Holomycota |  | *Aspergillus oryzae* |  |  |  |  |
|  |  |  |  | *Encephalitozoon cuniculi* |  |  |  |  |
|  |  |  |  | *Komagataella phaffii* |  | 1 |  |  |
|  |  |  |  | *Salpingoeca rosetta* |  |  |  |  |
|  |  |  |  | *Scutellospora calospora* |  | 1 |  | 1 |
|  |  |  |  | *Gigaspora margarita* |  | 1 |  | 1 |
|  |  |  |  | *Glomus cerebriforme* |  | 1 |  | 1 |
|  |  |  |  | *Basidiobolus meristosporus* |  | 1 |  | 1 |
|  |  |  |  | *Linderina pennispora* |  | 1 |  | 1 |
|  |  |  |  | *Spizellomyces punctatus* |  | 1 |  |  |
|  | Breviata |  |  | *Pygsuia biforma* |  |  |  |  |
|  | Apusomonadida |  |  | *Amastigomonas* sp. |  |  |  |  |
|  |  |  |  | *Thecamonas trahens* |  | 1 |  | 1 |
| CRuMs |  |  |  | *Diphylleia rotans* |  | 1 |  | 1 |
|  |  |  |  | *Mantamonas plastica* |  |  |  |  |
|  |  |  |  | *Rigifila ramosa* |  |  |  |  |
| Ancyromonadida |  |  |  | *Ancyromonas sigmoides* |  |  |  |  |
|  |  |  |  | *Fabomonas tropica* | 1 |  |  |  |
|  |  |  |  | *Nutomonas longa* |  |  |  |  |
| Discoba | Jakobid |  |  | *Andalucia incarcerata* |  | 1 |  | 1 |
|  |  |  |  | *Ophirina amphinema* | 1 |  | 1 |  |
|  | Heterolobosea |  |  | Eukaryote sp. BB2 | 1 |  |  |  |
|  |  |  |  | *Naegleria gruberi* |  |  |  |  |
|  | Tsukubea |  |  | *Tsukubamonas globosa* |  |  |  |  |
|  | Euglenozoa |  |  | *Euglena gracilis* |  |  |  |  |
|  |  |  |  | *Perkinsela sp.* |  |  |  |  |
|  |  |  |  | *Diplonema sp.* | 1 | 1 | 1 | 1 |
|  |  |  |  | *Trypanosoma cruzi* |  |  |  |  |
| Metamonada | Fornicata |  |  | *Chilomastix cuspidata* |  |  |  |  |
|  |  |  |  | *Dysnectes brevis* |  |  |  |  |
|  |  |  |  | *Kipferlia bialata* |  |  |  |  |
|  |  |  |  | *Barthelona* sp. PAP020 |  |  |  |  |
|  |  |  |  | *Giardia intestinalis* |  |  |  |  |
|  |  |  |  | *Trimastix marina* |  |  |  |  |
|  | Parabaslia |  |  | *Trichomonas vaginalis* |  |  |  |  |
|  |  |  |  | *Tritrichomonas foetus* |  |  |  |  |
| Malawimonas |  |  |  | *Malawimonas jakobiformis* |  | 1 |  | 1 |

The sequences that ware newly detected in this study were indicated in red.

The sequence labelled by a single asterisk (*) was assigned as ADAR in the phylogenetic analysis of the initial dataset (94 amino acids), but reassigned as ADAT in the phylogenetic analysis of the main dataset (209 amino acids).

Th inconsistence between the results of the initial dataset and the main dataset is probably due to low phylogenetic resolution in the small sized dataset (i.e., the initial dataset, 94 amino acids), therefore, the assignment of ADAR/ADAT in the initial dataset tree is not yet conclusive.
