# Supplementary material for: Massive RNA Editing in Ascetosporean Mitochondria
Source: Microbes Environ. 2025 Mar 15;40(1):ME24070. doi: 10.1264/jsme2.ME24070 (PMC11946409; doi:10.1264/jsme2.ME24070)
Supplement: Supplementary file 3 — Supplementary Material 3 [file 40_24070_s3.pdf]

SUPPLEMENTARY INFORMATION

Supplementary materials and methods

Scanning electron microscopy

Axenic cells grown on a glass slide were fixed with 2.5% glutaraldehyde in the cultivation medium at 4 °C. Fixed cells were washed using 0.22 µm filtered artificial seawater (FASW, 3.5% Rei-Sea Marine II; Iwaki Co. Ltd., Tokyo, Japan) and postfixed with 2% osmium tetroxide dissolved in FASW for 2 h. Postfixed cells were dehydrated using a graded ethanol series, dried using a JCPD-5 critical point drying device (JEOL, Akishima, Japan), and coated with osmium using an OPC-80 osmium coater (Filgen, Nagoya, Japan). The specimens were imaged using a field-emission scanning electron microscope (Quanta 450 FEG; Thermo Fisher Scientific) operating at 5 kV.

Western blot analysis

Approximately 200 mL mid-exponential-phase cell culture of Paradinida sp. FC901 was centrifuged at 2,400 × *g* for 5 min. The cell pellet was frozen and sent to Genostuff Co., Ltd. (Tokyo, Japan), where the following experiments were conducted.

The frozen cell pellet was homogenized in RIPA buffer (Fujifilm, Osaka, Japan) containing 1/100 (v/v) Protease Inhibitor Cocktail (Merck) for 30 min at 4 °C. After centrifugation at 18,000 × *g* for 5 min at 4 °C, the aqueous phase was recovered and utilized for initial protein assay using a BCA Protein Assay Kit (Thermo Fisher Scientific). Proteins (30 µg) were mixed with a sample buffer (Thermo Fisher Scientific) and a Sample Reducing Agent (Thermo Fisher Scientific) and separated by 5–20% gradient polyacrylamide gel electrophoresis. Separated proteins were transferred onto a polyvinylidene difluoride membrane (ATTO, Tokyo, Japan), and the membrane was blocked for 1 h at room temperature with 5% skim milk in Tris-buffered saline containing 0.1% Tween-20 (TBST). The membrane was then incubated overnight at 4 °C with a polyclonal anti-ADAR antibody HPA051519 (Merck) diluted to 1:500 in 0.1% TBST containing 5% bovine serum albumin. The

membrane was washed four times with 0.1% TBST and incubated for 1 h at room temperature with horseradish peroxidase-conjugated anti-rabbit IgG (#7074; Cell Signaling Technology, Danvers, MA, USA) diluted 1:5,000 with 0.05% TBST containing 5% skimmed milk. After washing the membrane four times with 0.05% TBST, immunoreactive bands were visualized using Immobilon (Merck) and recorded on a C-DiGit (LI-COR, Lincoln, NE, USA).

Supplementary discussion

Taxonomy of Paradinida spp. FC901 and SRM-001

Order Paradinida (paradinids) currently includes only one genus, *Paradinium* Chatton, 1910 (Chatton, 1910), and three species (Chatton and Soyer, 1973). As all the described species are parasites of copepods, several unidentified species that are possibly new have been reported from different copepod species (Chatton, 1920; Skovgaard and Daugbjerg, 2008). Although their complete life cycles are not understood, they have been estimated based on the findings of partial cell stages. Amoeboid cells grow in the body cavity of host copepods, and dividing cells are interconnected by fine pseudopodia. After growth in the body cavity, cells move to the lumen of the host intestine and are excreted through the anus. Released cells form an outstanding cyst, called a gonosphere, at the urosome of the host. Characteristics of the gonosphere, such as color, size, and number of chromosomes, are mainly utilized in their taxonomy (Chatton, 1910; Chatton and Soyer, 1973). Flagellated cells hatch from the gonosphere and probably re-parasitize another host; however, this has not yet been confirmed and the possibility of a secondary host has not been ruled out.

Cultures of FC901 and SRM-001 were established from water samples, not from copepods. Both strains grew axenically in Hemi medium and showed network-forming amoeboid cells (Figs. S1, S2). No other stages, such as flagellates or cysts, were observed. Amoeboid cells were similar to those of paradinids, and they phylogenetically branched within the Paradinida clade with high

statistical support. However, as gonospheres were not observed, and their characteristics could not be compared with those of the described species, their taxonomic identification remains unclear. FC901 and SRM-001 were phylogenetically distinct in the clade of Paradinida, and their morphological characteristics were slightly different; almost all FC901 cells had microvilli-like structures on the surface, whereas they were rarely observed in SRM-001 cells. This difference may be useful for distinguishing between them, although cells with and without microvilli-like structures were also found in the cultures of FC901 and SRM-001 (Figs. S1G, S2F). Therefore, we conclude that their proper identification and taxonomic discussion to distinguish them should be revisited with further data, and at present, they should be described only as Paradinida sp. FC901 and SRM-001.

References

Chatton, E. (1910) Protozoaires parasites des branchies des labres. *Amoeba Mucicola* 239–266.

Chatton, E. (1920) Les Péridiniens parasites: morphologie, reproduction, ethologie. *Arch Zool Exp Gén* **59**, 1–475

Chatton, E., and Soyer, M. (1973) Le cycle évolutif de *Paradinium poucheti* Chatton, flagellé parasite plasmodial des copépodes les paradinides. *Ann Sci Nat Zool* **12**: 27–60.

Flati, T., Gioiosa, S., Spallanzani, N., Tagliaferri, I., Diroma, M.A., Pesole, G., *et al.* (2020) HPC-REDItools: a novel HPC-aware tool for improved large scale RNA-editing analysis. *BMC Bioinformatics* **21(Suppl 10)**: 353.

Kim, D., Paggi, J.M., Park, C., Bennett, C., and Salzberg, S.L. (2019) Graph-based genome alignment and genotyping with HISAT2 and HISAT-genotype. *Nat Biotechnol* **37**: 907–915.

Skovgaard, A., and Daugbjerg, N. (2008) Identity and systematic position of *Paradinium poucheti* and other Paradinium-like parasites of marine copepods based on morphology and nuclear-encoded SSU rDNA. *Protist* **159**: 401–413.

Zhang, Z. (2022) KaKs_Calculator 3.0: calculating selective pressure on coding and non-coding sequences. *Genomics Proteomics Bioinformatics* **20**: 536–540.

Supplementary figures

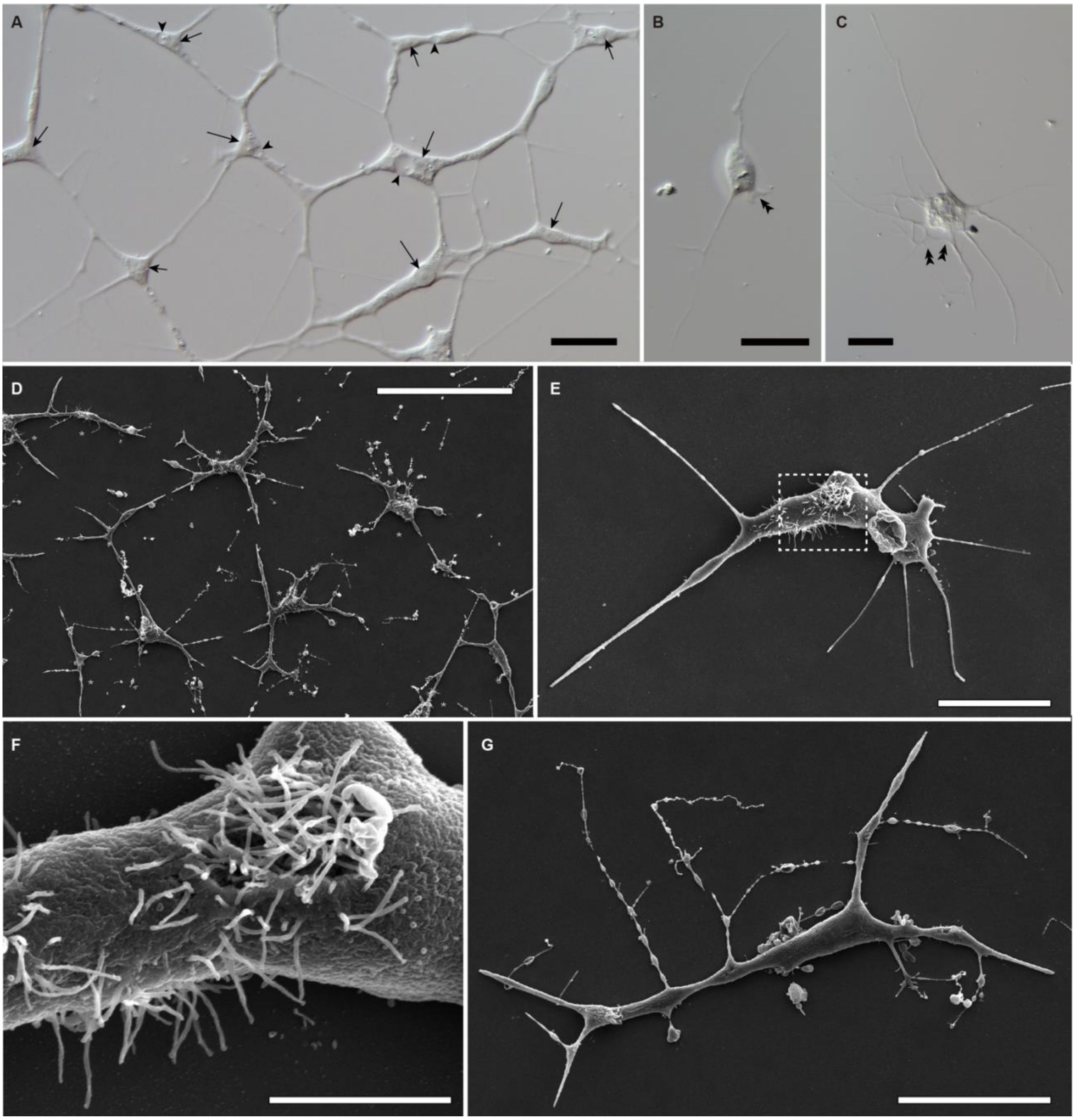

Fig. S1. Images of Paradinida sp. FC901 cells in the mid-exponential phase. **A.** Differential interference contrast (DIC) image of the network-forming cells. Each cell containing a single nucleus (arrow) is connected to the neighboring cells with thin filopodia. A clear vacuole (arrowhead) occasionally exists in cells. Scale bar, 20 µm. **B and C.** DIC images of the cell with a lobate

pseudopodium (double arrowhead). Scale bar, 20 µm. **D.** Scanning electron microscopic (SEM) image of network-forming cells. The connecting filopodia were broken by the fixation artifact. Cells marked by asterisks possess microvilli-like structures on the surface. Scale bar, 50 µm. **E.** High-magnification SEM image of the cell with the microvilli-like structure. Scale bar, 10 µm. **F.** Enlarged view of the microvilli-like structure enclosed by a white square in Fig. S1D. Scale bar, 3 µm. **G.** High-magnification SEM image of the cell without microvilli-like structure. Scale bar, 20 µm.

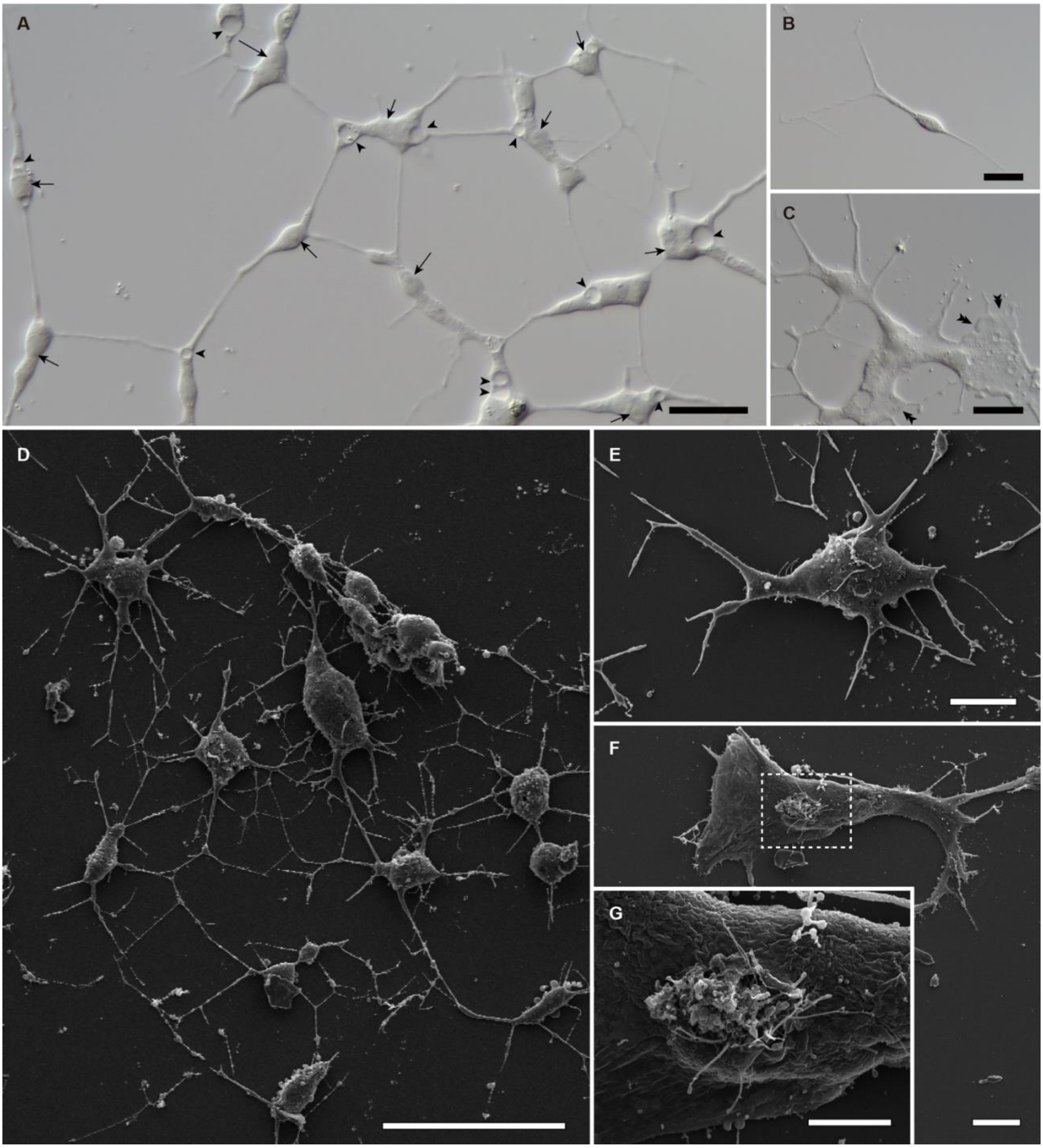

Fig. S2. Images of Paradinida sp. SRM-001 cells in the mid-exponential phase. **A.** DIC image of network-forming cells. Each cell containing a single nucleus (arrow) is connected with neighboring cells through thin filopodia. A clear vacuole (arrowhead) occasionally exists in cells. Scale bar, 20 µm. **B and C.** DIC images of the cell with a lobate pseudopodium (double arrowhead). Scale bar, 20

µm. **D.** SEM image of network-forming cells. Cells possessing microvilli-like structures on the surface are rarely recognized. Scale bar, 50 µm. **E.** High-magnification SEM image of the cell. Scale bar, 10 µm. **F.** High-magnification SEM image of the cell with microvilli-like structure. Scale bar, 20 µm. **G.** Enlarged view of the microvilli-like structure enclosed by a white square in Fig. S2F. Scale bar, 3 µm.

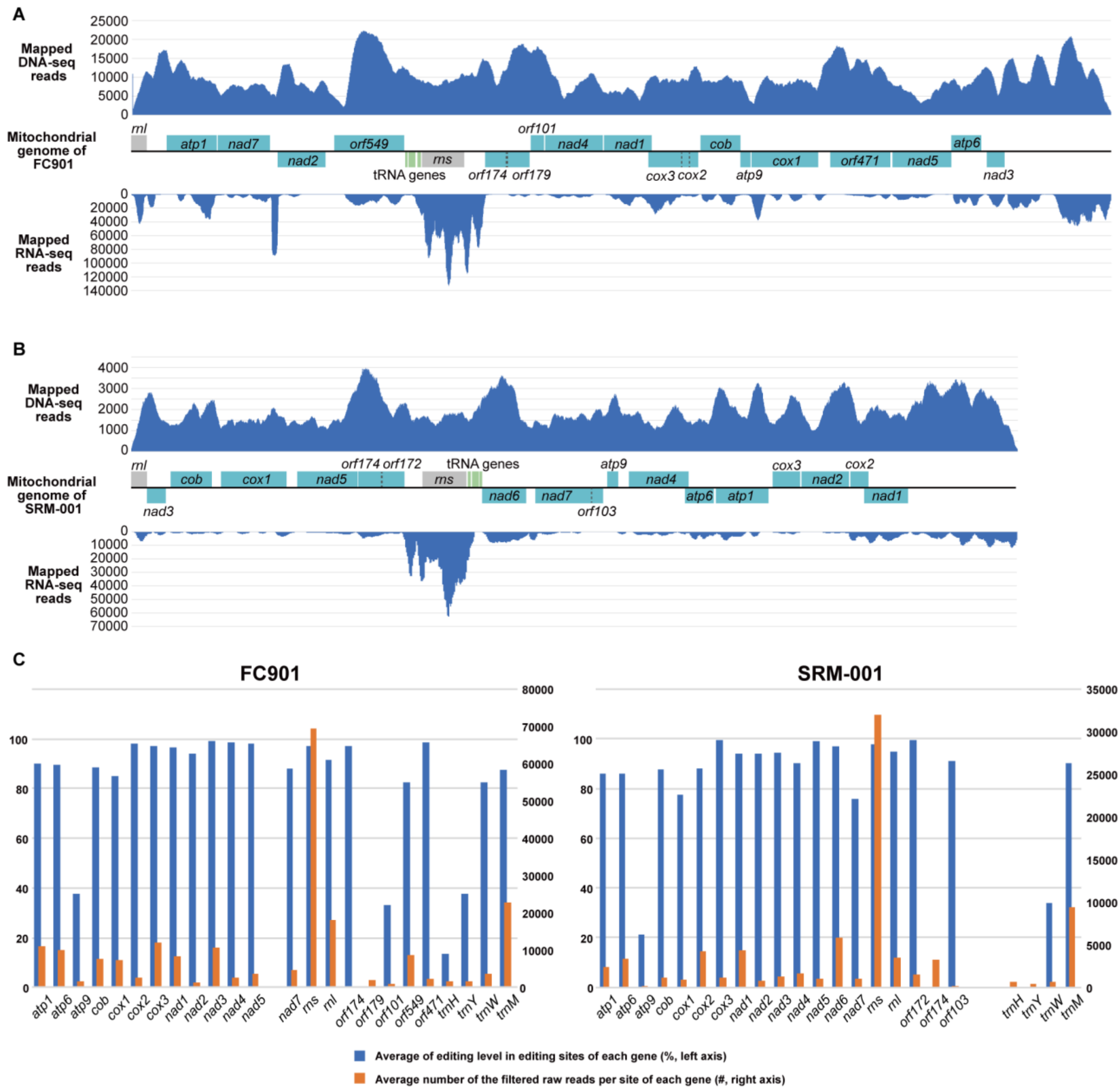

Fig. S3. Additional information about the mitochondrial genome reconstruction, and editing and transcriptional level of mitochondrial genes. **A, B.** Mapping results of the filtered reads of DNA-seq and RNA-seq data on the mitochondrial genome of FC901(A) and SRM-001 (B). **C.** Bar graphs summarizing the editing level and mapped read number of each gene.

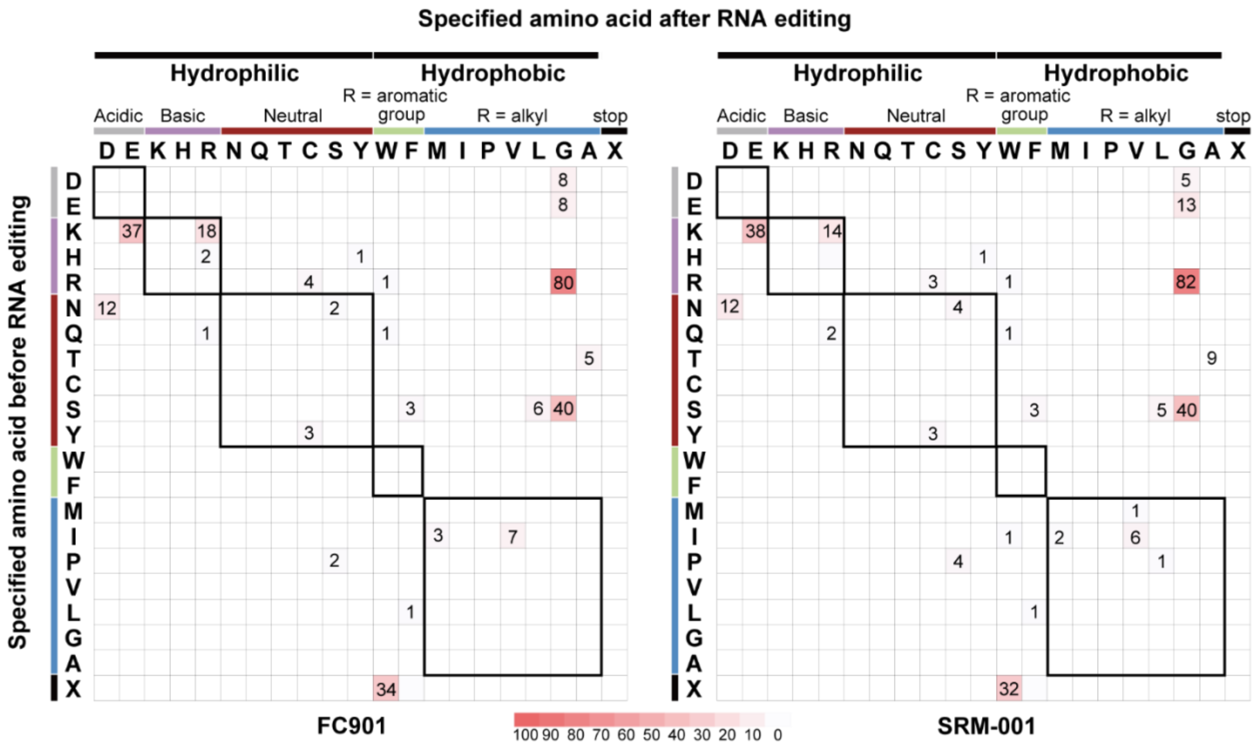

Fig. S4. Specified amino acids before and after RNA editing (editing level > 50%) in two paradinids. The number in each square indicates the number of the detected substitutions, which are also shown with heatmap color. Amino acids are represented by single letters. Substitutions in the same chemical groups are shown in bold-line squares.

Group	Organism	COX1	COB
Amorphea	*Homo sapiens*	YSRNHMFNPNWTYPLSNTTPDGDHLFWGHPEVPFGSMYFIPTKFFGGGHDHFHVSAFHGRRG	QGRHHGFGYMSWVTHHDPELKG
Amorphea	*Drosophila melanogaster*	YSRNHMFNPNWTYPLSNTTPDGDHLFWGHPEVPFGSMYFIPTKFFGGGHDHFHVSAFHGRRG	QGRHHGFGYMSWVTHHDPELKG
Amorphea	*Monosiga brevicollis* ATCC 50154	YSRNHMFNPNWTYPLSNTTPDGDHLFWGHPEVPFGSMYFIPTKFFGGGHDHFHVSAFHGRRG	QGRHHGFGYMSWVTHHDPELKG
Amorphea	*Saccharomyces castellii*	YSRNHMFNPNWTYPLSNTTPDGDHLFWGHPEVPFGSMYFIPTKFFGGGHDHFHVSAFHGRRG	QGRHHGFGYMSWVTHHDPELKG
Amorphea	*Candida parapsilosis*	YSRNHMFNPNWTYPLSNTTPDGDHLFWGHPEVPFGSMYFIPTKFFGGGHDHFHVSAFHGRRG	QGRHHGFGYMSWVTHHDPELKG
Amorphea	*Thecamonas trahens*	YSRNHMFNPNWTYPLSNTTPDGDHLFWGHPEVPFGSMYFIPTKFFGGGHDHFHVSAFHGRRG	QGRHHGFGYMSWVTHHDPELKG
Amorphea	*Dictyostelium discoideum*	YSRNHMFNPNWTYPLSNTTPDGDHLFWGHPEVPFGSMYFIPTKFFGGGHDHFHVSAFHGRRG	QGRHHGFGYMSWVTHHDPELKG
Amorphea	*Diphylleia rotans*	YSRNHMFNPNWTYPLSNTTPDGDHLFWGHPEVPFGSMYFIPTKFFGGGHDHFHVSAFHGRRG	QGRHHGFGYMSWVTHHDPELKG
	Malawimonas jakobiformis	YSRNHMFNPNWTYPLSNTTPDGDHLFWGHPEVPFGSMYFIPTKFFGGGHDHFHVSAFHGRRG	QGRHHGFGYMSWVTHHDPELKG
	Ancyromonas sigmoides	YSRNHMFNPNWTYPLSNTTPDGDHLFWGHPEVPFGSMYFIPTKFFGGGHDHFHVSAFHGRRG	QGRHHGFGYMSWVTHHDPELKG
Archaeplastida	*Ostreococcus tauri*	YSRNHMFNPNWTYPLSNTTPDGDHLFWGHPEVPFGSMYFIPTKFFGGGHDHFHVSAFHGRRG	QGRHHGFGYMSWVTHHDPELKG
Archaeplastida	*Nephroselmis olivacea*	YSRNHMFNPNWTYPLSNTTPDGDHLFWGHPEVPFGSMYFIPTKFFGGGHDHFHVSAFHGRRG	QGRHHGFGYMSWVTHHDPELKG
Archaeplastida	*Arabidopsis thaliana*	YSRNHMFNPNWTYPLSNTTPDGDHLFWGHPEVPFGSMYFIPTKFFGGGHDHFHVSAFHGRRG	QGRHHGFGYMSWVTHHDPELKG
Archaeplastida	*Mesostigma viride*	YSRNHMFNPNWTYPLSNTTPDGDHLFWGHPEVPFGSMYFIPTKFFGGGHDHFHVSAFHGRRG	QGRHHGFGYMSWVTHHDPELKG
Archaeplastida	*Picobiliphyte* sp. MS584-11	YSRNHMFNPNWTYPLSNTTPDGDHLFWGHPEVPFGSMYFIPTKFFGGGHDHFHVSAFHGRRG	QGRHHGFGYMSWVTHHDPELKG
Archaeplastida	*Porphyra purpurea*	YSRNHMFNPNWTYPLSNTTPDGDHLFWGHPEVPFGSMYFIPTKFFGGGHDHFHVSAFHGRRG	QGRHHGFGYMSWVTHHDPELKG
Archaeplastida	*Cyanidioschyzon merolae*	YSRNHMFNPNWTYPLSNTTPDGDHLFWGHPEVPFGSMYFIPTKFFGGGHDHFHVSAFHGRRG	QGRHHGFGYMSWVTHHDPELKG
Archaeplastida	*Chondrus crispus*	YSRNHMFNPNWTYPLSNTTPDGDHLFWGHPEVPFGSMYFIPTKFFGGGHDHFHVSAFHGRRG	QGRHHGFGYMSWVTHHDPELKG
Archaeplastida	*Cyanophora paradoxa*	YSRNHMFNPNWTYPLSNTTPDGDHLFWGHPEVPFGSMYFIPTKFFGGGHDHFHVSAFHGRRG	QGRHHGFGYMSWVTHHDPELKG
Pancryptista	*Microheliella maris*	YSRNHMFNPNWTYPLSNTTPDGDHLFWGHPEVPFGSMYFIPTKFFGGGHDHFHVSAFHGRRG	QGRHHGFGYMSWVTHHDPELKG
Pancryptista	*Palpitomonas bilix*	YSRNHMFNPNWTYPLSNTTPDGDHLFWGHPEVPFGSMYFIPTKFFGGGHDHFHVSAFHGRRG	QGRHHGFGYMSWVTHHDPELKG
Pancryptista	*Leucocryptos marina*	YSRNHMFNPNWTYPLSNTTPDGDHLFWGHPEVPFGSMYFIPTKFFGGGHDHFHVSAFHGRRG	QGRHHGFGYMSWVTHHDPELKG
Pancryptista	*Guillardia theta*	YSRNHMFNPNWTYPLSNTTPDGDHLFWGHPEVPFGSMYFIPTKFFGGGHDHFHVSAFHGRRG	QGRHHGFGYMSWVTHHDPELKG
Pancryptista	*Rhodomonas salina*	YSRNHMFNPNWTYPLSNTTPDGDHLFWGHPEVPFGSMYFIPTKFFGGGHDHFHVSAFHGRRG	QGRHHGFGYMSWVTHHDPELKG
Haptista	*Emiliania huxleyi*	YSRNHMFNPNWTYPLSNTTPDGDHLFWGHPEVPFGSMYFIPTKFFGGGHDHFHVSAFHGRRG	QGRHHGFGYMSWVTHHDPELKG
Haptista	*Diacronema lutheri*	YSRNHMFNPNWTYPLSNTTPDGDHLFWGHPEVPFGSMYFIPTKFFGGGHDHFHVSAFHGRRG	QGRHHGFGYMSWVTHHDPELKG
Haptista	*Marophrys* sp. SRT127	YSRNHMFNPNWTYPLSNTTPDGDHLFWGHPEVPFGSMYFIPTKFFGGGHDHFHVSAFHGRRG	QGRHHGFGYMSWVTHHDPELKG
	Ancoracysta twista	YSRNHMFNPNWTYPLSNTTPDGDHLFWGHPEVPFGSMYFIPTKFFGGGHDHFHVSAFHGRRG	QGRHHGFGYMSWVTHHDPELKG
	Meteora sporadica	YSRNHMFNPNWTYPLSNTTPDGDHLFWGHPEVPFGSMYFIPTKFFGGGHDHFHVSAFHGRRG	QGRHHGFGYMSWVTHHDPELKG
SAR	*Thalassiosira pseudonana*	YSRNHMFNPNWTYPLSNTTPDGDHLFWGHPEVPFGSMYFIPTKFFGGGHDHFHVSAFHGRRG	QGRHHGFGYMSWVTHHDPELKG
SAR	*Ectocarpus siliculosus*	YSRNHMFNPNWTYPLSNTTPDGDHLFWGHPEVPFGSMYFIPTKFFGGGHDHFHVSAFHGRRG	QGRHHGFGYMSWVTHHDPELKG
SAR	*Nannochloropsis limnetica*	YSRNHMFNPNWTYPLSNTTPDGDHLFWGHPEVPFGSMYFIPTKFFGGGHDHFHVSAFHGRRG	QGRHHGFGYMSWVTHHDPELKG
SAR	*Ochromonas danica*	YSRNHMFNPNWTYPLSNTTPDGDHLFWGHPEVPFGSMYFIPTKFFGGGHDHFHVSAFHGRRG	QGRHHGFGYMSWVTHHDPELKG
SAR	*Cafeteria roenbergensis*	YSRNHMFNPNWTYPLSNTTPDGDHLFWGHPEVPFGSMYFIPTKFFGGGHDHFHVSAFHGRRG	QGRHHGFGYMSWVTHHDPELKG
SAR	*Phytophthora sojae*	YSRNHMFNPNWTYPLSNTTPDGDHLFWGHPEVPFGSMYFIPTKFFGGGHDHFHVSAFHGRRG	QGRHHGFGYMSWVTHHDPELKG
SAR	*Plasmodium falciparum*	YSRNHMFNPNWTYPLSNTTPDGDHLFWGHPEVPFGSMYFIPTKFFGGGHDHFHVSAFHGRRG	QGRHHGFGYMSWVTHHDPELKG
SAR	*Theileria parva*	YSRNHMFNPNWTYPLSNTTPDGDHLFWGHPEVPFGSMYFIPTKFFGGGHDHFHVSAFHGRRG	QGRHHGFGYMSWVTHHDPELKG
SAR	*Bigelowiella natans*	YSRNHMFNPNWTYPLSNTTPDGDHLFWGHPEVPFGSMYFIPTKFFGGGHDHFHVSAFHGRRG	QGRHHGFGYMSWVTHHDPELKG
SAR	*Spongospora subterranea*	YSRNHMFNPNWTYPLSNTTPDGDHLFWGHPEVPFGSMYFIPTKFFGGGHDHFHVSAFHGRRG	QGRHHGFGYMSWVTHHDPELKG
Discoba	*Reclinomonas americana*	YSRNHMFNPNWTYPLSNTTPDGDHLFWGHPEVPFGSMYFIPTKFFGGGHDHFHVSAFHGRRG	QGRHHGFGYMSWVTHHDPELKG
Discoba	*Andalucia godoyi*	YSRNHMFNPNWTYPLSNTTPDGDHLFWGHPEVPFGSMYFIPTKFFGGGHDHFHVSAFHGRRG	QGRHHGFGYMSWVTHHDPELKG
Discoba	*Bodo saltans*	YSRNHMFNPNWTYPLSNTTPDGDHLFWGHPEVPFGSMYFIPTKFFGGGHDHFHVSAFHGRRG	QGRHHGFGYMSWVTHHDPELKG
Discoba	*Trypanoplasma borreli*	YSRNHMFNPNWTYPLSNTTPDGDHLFWGHPEVPFGSMYFIPTKFFGGGHDHFHVSAFHGRRG	QGRHHGFGYMSWVTHHDPELKG
Discoba	*Naegleria fowleri*	YSRNHMFNPNWTYPLSNTTPDGDHLFWGHPEVPFGSMYFIPTKFFGGGHDHFHVSAFHGRRG	QGRHHGFGYMSWVTHHDPELKG
Discoba	*Pharyngomonas kirbyi*	YSRNHMFNPNWTYPLSNTTPDGDHLFWGHPEVPFGSMYFIPTKFFGGGHDHFHVSAFHGRRG	QGRHHGFGYMSWVTHHDPELKG
Discoba	*Euglena gracilis*	YSRNHMFNPNWTYPLSNTTPDGDHLFWGHPEVPFGSMYFIPTKFFGGGHDHFHVSAFHGRRG	QGRHHGFGYMSWVTHHDPELKG
Discoba	*Hemistasia phaeocysticola*	YSRNHMFNPNWTYPLSNTTPDGDHLFWGHPEVPFGSMYFIPTKFFGGGHDHFHVSAFHGRRG	QGRHHGFGYMSWVTHHDPELKG
Discoba	*Diplonema papillatum*	YSRNHMFNPNWTYPLSNTTPDGDHLFWGHPEVPFGSMYFIPTKFFGGGHDHFHVSAFHGRRG	QGRHHGFGYMSWVTHHDPELKG
Discoba	*Rhynchopus euleeides*	YSRNHMFNPNWTYPLSNTTPDGDHLFWGHPEVPFGSMYFIPTKFFGGGHDHFHVSAFHGRRG	QGRHHGFGYMSWVTHHDPELKG
Discoba	*Tsukubamonas globosa*	YSRNHMFNPNWTYPLSNTTPDGDHLFWGHPEVPFGSMYFIPTKFFGGGHDHFHVSAFHGRRG	QGRHHGFGYMSWVTHHDPELKG
	***Bonamia ostreae* DNA**	**-----MFNPNWTYPLSNTTPDGDHLFWGHPEVPFGSMFFIPTKLFGGGHDHFHVAIFHGRRG**	**--KHHGFGSISWVVHHFPSWKG**
	**FC901 DNA**	**YSKNHMSNPNxTYPLSNTSPDRDHLFxDHPKVPFRTMYFIPTKFFEESHNHFHVSAFHDHRS**	**QRRHHRFSYMPxITHHNPESKR**
	**SRM-001 DNA**	**YSKNHMSNPNxTYPLSNTSPDRDHLFxEHPKMPFSTMYFIPTKFFEERHNHSHVSAFHEKRR**	**QSRHHRFRYMSxVTHHNPEPKR**
	**FC901 RNA (50% > EL)**	**YSRNHMFNPNWTYPLSNTSPDGDHLFWGHPEVPFGTMYFIPTKFFGGGHDHFHVSAFHGRRG**	**QGRHHGFGYMPxVTHHDPELKG**
	**SRM-001 RNA (50% > EL)**	**YSRNHMFNPNxTYPLSNTSPDGDHLFWGHPKMPFGTMYFIPTKFFGGGHDHSHVSAFHGRRG**	**QGRHHGFGYMSWVTHHDPELKG**
		W; EL = 40% · E; EL = 35% · V; EL = 38% · F; EL = 35%	★ S; EL = 1% · W; EL = 43%

Fig. S5. The alignment of well-conserved amino acid (AA) residues in COX1 and COB sequences. The 62 and 22 AA residues shown here were completely conserved in whole eukaryotes except for ascetosporeans in our alignments of COX1 and COB, respectively. The mismatched AAs and the stop codons (x) detected in ascetosporean DNA and RNA sequences are shown in red. The AAs shown in green indicated that they were restored to the conserved AAs by RNA editing. Two positions in FC901 COB and four positions in SRM-001 COX1, respectively, were not restored when 50% of editing level (EL) was applied as the threshold. However, five of them were also restored at approximately 40% of editing level. Their editing levels were specifically shown in gray squares. At only one position in FC901 COB (marked with a red star), the editing level corresponding to the AA restoration was 1%. The AA residues that were estimated from the candidate of the partial mitochondrial genome of *Bonamioa ostreae* (Haplosporida) with translation codon table 4 (i.e., table for mold, protozoan, and coelenterate mitochondrial + *Mycoplasma* / *Spiroplasma*) added.

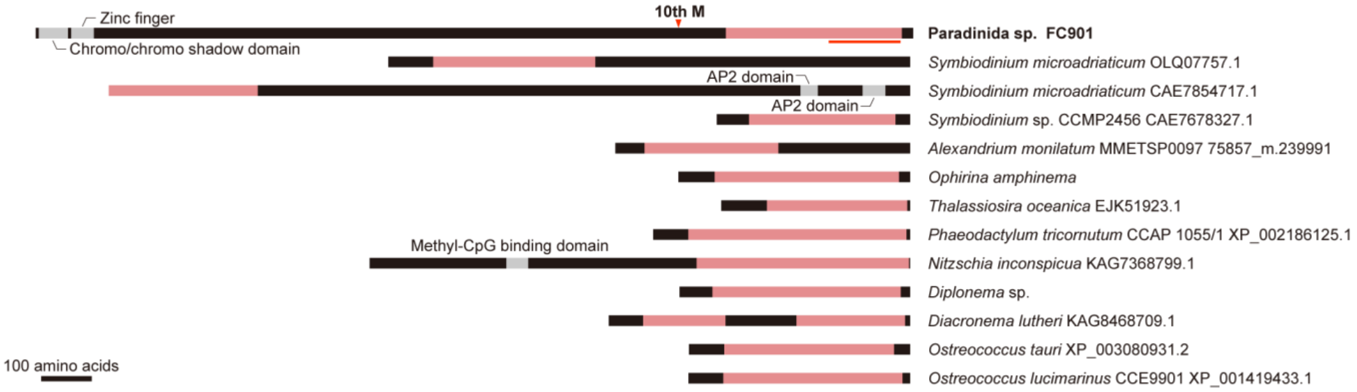

Fig. S6. Domain structures of the selected ADAR-like sequences. Pink bars indicate deaminase domains, with other detected domains indicated by dark grey bars. ADAR-like sequence of SRM-001 was very partial and is not shown here. The position of 10th methionine is shown with a red arrowhead and antigenic region of anti-ADAR antibody HPA051519 are shown with a red bar.

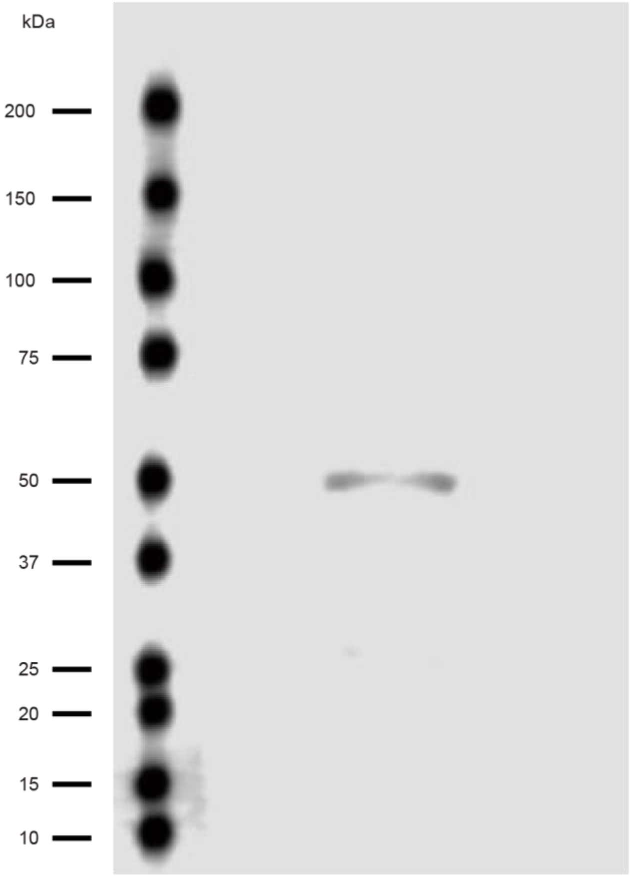

Fig. S7. Western blot analysis of anti-ADAR antibody HPA051519 with Paradinida sp. FC901, with a single band at approximately 50 kDa.

Supplementary tables

Table S1. Summary of the initial samples for establishing the cultures. The location, longitude, latitude, depth (m) and date are given for both cultures.

	Sampling area	Longitude (N)	Latitude (E)	Depth (m)	Date
Paradinida sp. FC901	Tokyo Bay, Japan	35.3194°	139.6507°	Surface	November 16, 2019
Paradinida sp. SRM-001	Suruga Bay, Japan	34.8833°	138.6417°	200	January 15, 2019

Table S2. Summary of the sequencing analyses. The DRR number, the DNA and RNA extraction kits, the library kit, sequencing target, platform, reads, bases, Q30 (%), number of contigs, and BUSCO (%) are given for each.

Organism	DRR no.	DNA/RNA Extraction kit	Library kit	Sequencing target	Platform	Reads	Bases (Gb)	Q30 (%)	No. of contigs	BUSCO (%)
Paradinida sp. SRM-001	DRR408102	Invitrogen PureLink Genomic DNA Mini Kit	VAHTS Universal Pro DNA Library Prep Kit for Illumina	Genomic DNA	NovaSeq 6000	51,617,946	15.485	88.98	-	-
	DRR408103	Qiagen RNeasy Mini Kit	NEBNext Ultra II Directional RNA Library Prep Kit for Illumina	mRNA	NovaSeq 6000	13,073,696	3.922	93.95	86,986	62.7
Paradinida sp. FC901	DRR408104	Invitrogen PureLink Genomic DNA Mini Kit	VAHTS Universal Pro DNA Library Prep Kit for Illumina	Genomic DNA	NovaSeq 6000	39,678,757	11.904	92.64	-	-
	DRR408105	TRI Reagent, followed by RNA Clean & Concentrator Kits	NEBNext Ultra II Directional RNA Library Prep Kit for Illumina	mRNA	NovaSeq 6000	13,595,734	4.079	94.52		
	DRR408106	Qiagen RNeasy Mini Kit	MGIEasy RNA Directional Library Prep Set V2.0	mRNA	DNBSEQ-G400	78,445,090	11.766	85.44	178,021	85.1
	DRR408107	TRI Reagent, followed by RNA Clean & Concentrator Kits	NEBNext Ultra II Directional RNA Library Prep Kit for Illumina	mRNA	NovaSeq 6000	193,727,174	58.118	94.16		

Table S3. Summary of the site number of each DNA heterogeneity in the mitochondrial genome of two paradinids. The rate of heterogeneity (%) and the number of sites are given for both samples.

Strain	Rate of heterogeneity (%)	Number of sites
FC901	26.6	1
	7.3	1
	2≥, >1	4
	1≥, >0	4
	0	23,038 (99.96%)
	Total	23,048
SRM-001	7.0	1
	4≥, >3	1
	3≥, >2	2
	2≥, >1	29
	1≥, >0	175
	0	19,891 (98.97%)
	Total	20,099

Table S4. Summary of mitochondrial RNA editing in Paradinida spp. FC901 and SRM-001.

Shown in separate excel file.

The number of the editing sites was counted applying the threshold to more than 50%, 30% or 10%. The highest and second highest editing density., i.e., the number of editing sites per gene length, are shown on orange and pale orange backgrounds in FC901 and green and pale green backgrounds, respectively.

* The editing density is calculated using the following formula:

$$\text{Editing density} = \frac{\text{number of editing sites in a given gene} \times 100}{\text{length of a given gene}}$$

Table S5. Number of the editing sites at each codon position per level.

Editing level (%)	FC901			SRM-001		
	codon1	codon2	codon3	codon1	codon2	codon3
100≥, >90	190	71	4	165	56	2
90≥, >80	8	13	1	20	13	0
80≥, >70	7	5	0	7	7	0
70≥, >60	5	4	0	4	3	0
60≥, >50	5	1	0	6	5	0
50≥, >40	3	4	0	5	0	0
40≥, >30	8	3	0	5	10	0
30≥, >20	5	1	0	6	6	1
20≥, >10	1	3	0	5	2	0
10≥, >0	33	40	50	34	31	53
0	5427	5547	5637	4510	4634	4711

Table S6. Summary of editing level and mapped read number of RNA-seq of each gene. The editing level average (%) and mapped read number average are given for each gene for FC901 and SRM-001.

Gene	FC901		SRM-001	
	Editing level average (%)*	Mapped read number average (#)**	Editing level average (%)*	mapped read number average (#)**
atp1	89.8	11067.3	85.8	2397.9
atp6	89.5	10008.0	85.9	3288.6
atp9	37.7	1470.9	21.0	84.0
cob	88.3	7540.8	87.5	1075.7
cox1	84.7	7380.6	77.6	880.5
cox2	98.1	2633.7	87.9	4230.0
cox3	96.9	12145.6	99.3	1130.7
nad1	96.3	8326.4	93.8	4287.6
nad2	94.0	1136.4	93.8	684.4
nad3	99.1	10724.5	94.6	1274.3
nad4	98.3	2708.9	90.3	1625.3
nad5	98.0	3667.0	98.9	958.2
nad6 (SRM-001 only)			97.0	5822.7
nad7	88.1	4534.6	75.9	964.2
rns	97.0	69410.4	97.9	31980.4
rnl	91.3	18058.2	94.6	3406.6
orf174 (*orf172* of SRM-001)	97.0	377.9	99.7	1449.4
orf179 (*orf174* of SRM-001)	NA	2102.6	NA	3268.3
orf101 (*orf103* of SRM-001)	33.0	1066.1	91.0	164.1
orf549 (FC901 only)	82.3	8620.7		
orf471 (FC901 only)	98.3	2238.2		
trnH	13.5	1519.0	NA	620.4
trnY	37.5	1497.2	NA	406.0
trnW	82.6	3586.0	33.8	622.3
trnM	87.5	22887.0	90.0	9393.9
r	0.261613172		0.237952855	

t	1.271352174	1.122684155
p	0.216880991	0.274243922

'*r', 't', and 'p*' at the lowest three rows indicate the results of statistical analysis for correlation between editing level average and mapped read number average (= transcriptional level).

*The editing level average of each gene was calculated from the sites that were detected at more than 10% editing level threshold. Sites that were not involved in RNA editing were excluded from the analysis.

** The mapped read average is calculated using the following formula:

Mapping read average = number of total raw reads mapping on a given gene / length of a given gene.

Table S7. Results of Mitofates analyses.

Gene	Organism	contig ID or accession number	Probability of mitochondrial presequence
PPR-DYW	Paradinida sp. FC901	TRINITY_DN317_c0_g3_i1	0.717
	Crepidotus variabilis	KAF9528688.1	0.997
	Galerina marginata CBS 339.88	KDR72683.1	0.984
	Flammula alnicola	KAF8954993.1	0.983
	Hebeloma cylindrosporum h7	KIM46640.1	0.934
	Laccaria amethystina LaAM-08-1	KIK00860.1	0.87
	Amanita rubescens	KAF8337132.1	0.669
	Piloderma croceum F 1598	KIM77073.1	0.68
	Laccaria bicolor S238N-H82	XP_001886834.1	0.031
	Amanita phalloides	KAK2461236.1	0.285
PPR protein	Paradinida sp. FC901	TRINITY_DN11807_c1_g1_i1	1
	Paradinida sp. FC901	TRINITY_DN11442_c0_g1_i1	0.997
	Paradinida sp. FC901	TRINITY_DN7867_c1_g1_i6	0.994
	Paradinida sp. FC901	TRINITY_DN4182_c0_g1_i13	0.99
	Paradinida sp. FC901	TRINITY_DN4182_c0_g1_i15	0.99
	Paradinida sp. FC901	TRINITY_DN4182_c0_g1_i22	0.99
	Paradinida sp. FC901	TRINITY_DN4182_c0_g1_i4	0.99
	Paradinida sp. FC901	TRINITY_DN4182_c0_g1_i5	0.99
	Paradinida sp. FC901	TRINITY_DN4182_c0_g1_i9	0.99
	Paradinida sp. FC901	TRINITY_DN2700_c0_g2_i1	0.987
	Paradinida sp. FC901	TRINITY_DN62929_c0_g1_i1	0.961
	Paradinida sp. FC901	TRINITY_DN7867_c1_g1_i2	0.953
	Paradinida sp. FC901	TRINITY_DN4182_c0_g1_i20 *	0.566
	Paradinida sp. FC901	TRINITY_DN4182_c0_g1_i17 *	0.153
	Paradinida sp. FC901	TRINITY_DN7352_c0_g2_i1	0.058
	Paradinida sp. FC901	TRINITY_DN7352_c0_g1_i2	0.023
	Paradinida sp. FC901	TRINITY_DN2579_c4_g3_i1	0.013
	Paradinida sp. FC901	TRINITY_DN2579_c4_g1_i10	0.008
	Paradinida sp. FC901	TRINITY_DN4182_c0_g1_i19	0.008

	Paradinida sp. FC901	TRINITY_DN4182_c0_g1_i6	0.008
	Paradinida sp. FC901	TRINITY_DN2579_c4_g1_i16	0.006
	Paradinida sp. FC901	TRINITY_DN7352_c0_g1_i5	0.006
	Paradinida sp. FC901	TRINITY_DN7352_c0_g1_i7	0.006
	Paradinida sp. FC901	TRINITY_DN7352_c0_g1_i1	0.004
	Paradinida sp. FC901	TRINITY_DN10504_c0_g1_i1	0.003
	Paradinida sp. FC901	TRINITY_DN20122_c0_g1_i1	0.002
	Paradinida sp. FC901	TRINITY_DN20122_c0_g1_i2	0.002
	Paradinida sp. FC901	TRINITY_DN20122_c0_g1_i3	0.002
	Paradinida sp. FC901	TRINITY_DN2579_c4_g1_i11	0.002
	Paradinida sp. FC901	TRINITY_DN2579_c4_g1_i12	0.002
	Paradinida sp. FC901	TRINITY_DN2579_c4_g1_i14	0.002
	Paradinida sp. FC901	TRINITY_DN2579_c4_g1_i7	0.002
	Paradinida sp. FC901	TRINITY_DN2579_c4_g1_i9	0.002
	Paradinida sp. FC901	TRINITY_DN4182_c0_g1_i10	0.002
	Paradinida sp. FC901	TRINITY_DN4182_c0_g1_i16	0.002
	Paradinida sp. FC901	TRINITY_DN6977_c1_g2_i1 *	0.002
	Paradinida sp. FC901	TRINITY_DN7352_c0_g3_i2 *	0.002
	Paradinida sp. FC901	TRINITY_DN12304_c0_g1_i1	0.001
	Paradinida sp. FC901	TRINITY_DN4182_c0_g1_i18	0.001
	Paradinida sp. FC901	TRINITY_DN6977_c0_g1_i1	0.001
	Paradinida sp. FC901	TRINITY_DN6977_c1_g1_i1 *	0.001
	Paradinida sp. FC901	TRINITY_DN289_c3_g1_i3	0
ADAR-like	Paradinida sp. FC901	TRINITY_DN2269_c0_g1_i3	0.001
	Paradinida sp. FC901	TRINITY_DN2269_c0_g1_i3 (from 10th M)	0.104

In the sequences indicated with a single asterisk, a methionine was not detected in the first 50 amino acids and they may have been sequenced partially. Those sequences were directly subjected to MitoFates analysis without trimming the N´ region before first methionine.

PPR-DYW protein of *Hygrocybe coccinea* was found from its genome sequence and full length of its PPR-DYW protein could not be determined certainly. Its sequence was omitted from MitoFates analysis.

The scores with green and gray backgrounds are corresponding to positive and negative results of the existence of mitochondrial presequence, respectively.

Table S8. List of ADAR, ADAR-like, and ADAT sequences analyzed in this study.

					ADAR in the initial dataset tree	ADAT in the initial dataset tree	pADAR in the main dataset tree	ADAT in the main dataset tree
Diaphretickes	CAM clade	Archaeplastida	Viridiplantae	*Chlamydomonas reinhardtii*				
				Chlorokybus atmophyticus		1		1
				Mesostigma viride				
				Physcomitrium patens		1		1
				Ulva flexuosa				
				Ostreococcus lucimarinus	1		1	
				Ostreococcus tauri	1	1	1	
				Marchantia polymorpha		1		1
				Physcomitrium patens		1		1
				Papaver bracteatum		1		1
				Cypripedium flavum		1		1
				Coffea eugenioides		1		1
				Arabidopsis suecica		1		1
				Arabidopsis thaliana		1		1
				Prunus persica		1		1
				Ziziphus jujuba		1		1
				Syzygium oleosum		1		1
				Rhododendron griersonianum		1		1
			Rhodophyta	*Rhodelphis limneticus*	1			1*
				Rhodelphis marinus	1			1*
				Cyanidioschyzon merolae				
				Galdieria sulphuraria				
				Chondrus crispus				
			Glaucophyta	*Cyanophora paradoxa*				
		Pancryptista		*Microheliella maris*				
				Guillardia theta	1	1		

			Hemiarma marina	1			
			Goniomonas avonlea		1		
Telonema			*Telonema* sp. P-2	2			
SAR	Alveolata	Cilliate	*Oxytricha trifallax*				
			Paramecium caudatum				
		Dinoflagellate	*Pyrodinium bahamense*	1	1		1
			Scrippsiella trochoidea CCMP3099	2	2		
			Alexandrium monilatum CCMP3105	2	1	1	1
			Symbiodinium microadriaticum	2		2	
			Symbiodinium sp. CCMP2456	1	1	1	1
			Symbiodinium sp. CCMP2592		1		1
			Symbiodinium pilosum		1		1
		Apicomplexa	*Plasmodium falciparum*				
	Stramenopile		*Phaeodactylum tricornutum*	1	1	1	1
			Thalassiosira oceanica	1	1	1	1
			Nitzschia inconspicua	1	1	1	1
			Fistulifera solaris		1		1
			Blastocystis sp.		1		1
			Blastocystis hominis		1		1
			Phytophthora megakarya		1		1
			Albugo candida		1		1
	Rhizaria		*Bigelowiella natans*	1			
			Plasmodiophora brassicae				
			Paulinella sp.	1			
			Paradinium sp. FC901	1	1	1	
			Paradinium sp. SRM	1	1	1	
			Eucyrtidium acuminatum	1			
Haptophyta			*Chrysochromulina rotalis*	1			
			Emiliania huxleyi		2		

				Diacronema lutheri	1		1	
				Marophrys sp. SRT127		1		1
	Centrohelida			*Choanocystis* sp. FB-2015	1			
				Raineriophrys erinaceoides				
	Ancoracysta			*Ancoracysta twista*				
	Hemimastigophra			*Hemimastix kukwesjijk*				
				Spironema cf. multiciliatum				
				Acanthamoeba castellanii		1		1
	Amoebozoa			*Dictyostelium discoideum*		1		1
				Entamoeba histolytica		1		
				Capsaspora owczarzaki		1		1
				Abeoforma whisleri				
				Creolimax fragrantissima	2			1**
				Homo sapiens	3	1	3	1
				Mus musculus	3	1	3	1
		Holozoa		*Xenopus tropicalis*	3	1	3	1
			Metazoa	*Gallus gallus*	3	1	3	1
				Danio rerio	3	1	3	1
Amorphea				*Ciona intestinalis*	2	1	2	1
	Opisthokonts			*Strongylocentrotus purpuratus*	2	1	2	1
				Amphimedon queenslandica	2	1	2	1
				Aspergillus oryzae				
				Encephalitozoon cuniculi				
				Komagataella phaffii		1		
				Salpingoeca rosetta				
		Holomycota		*Scutellospora calospora*		1		1
				Gigaspora margarita		1		1
				Glomus cerebriforme		1		1
				Basidiobolus meristosporus		1		1
				Linderina pennispora		1		1

		Spizellomyces punctatus		1		
	Breviata	*Pygsuia biforma*				
	Apusomonadida	*Amastigomonas* sp.				
		Thecamonas trahens		1		1
CRuMs		*Diphylleia rotans*		1		1
		Mantamonas plastica				
		Rigifila ramosa				
Ancyromonadida		*Ancyromonas sigmoides*				
		Fabomonas tropica	1			
		Nutomonas longa				
Discoba	Jakobid	*Andalucia incarcerata*		1		1
		Ophirina amphinema	1		1	
	Heterolobosea	Eukaryote sp. BB2	1			
		Naegleria gruberi				
	Tsukubea	*Tsukubamonas globosa*				
	Euglenozoa	*Euglena gracilis*				
		Perkinsela sp.				
		Diplonema sp.	1	1	1	1
		Trypanosoma cruzi				
Metamonada	Fornicata	*Chilomastix cuspidata*				
		Dysnectes brevis				
		Kipferlia bialata				
		Barthelona sp. PAP020				
		Giardia intestinalis				
		Trimastix marina				
	Parabaslia	*Trichomonas vaginalis*				
		Tritrichomonas foetus				
Malawimonas		*Malawimonas jakobiformis*		1		1

The sequences that ware newly detected in this study were indicated in red.

The sequence labelled by a single asterisk (*) was assigned as ADAR in the phylogenetic analysis of the initial dataset (94 amino acids), but reassigned as ADAT in the phylogenetic analysis of the main dataset (209 amino acids).

Th inconsistence between the results of the initial dataset and the main dataset is probably due to low phylogenetic resolution in the small sized dataset (i.e., the initial dataset, 94 amino acids), therefore, the assignment of ADAR/ADAT in the initial dataset tree is not yet conclusive.
